# Individual Versus Combined Effects of Warming, Elevated CO_2_ and Drought on Grassland Water Uptake and Fine Root Traits

**DOI:** 10.1111/pce.15274

**Published:** 2024-11-18

**Authors:** Maud Tissink, Jesse Radolinski, David Reinthaler, Sarah Venier, Erich M. Pötsch, Andreas Schaumberger, Michael Bahn

**Affiliations:** ^1^ Department of Ecology Universität Innsbruck Innsbruck Austria; ^2^ Department of Environmental Science and Technology University of Maryland College Park Maryland USA; ^3^ Agricultural Research and Education Centre (AREC), Raumberg‐Gumpenstein Irdning Austria

**Keywords:** global change, grassland water dynamics, root traits, root water uptake

## Abstract

Increasing warming, atmospheric CO_2_ and drought are expected to change the water dynamics of terrestrial ecosystems. Yet, limited knowledge exists about how the interactive effects of these factors will affect grassland water uptake, and whether adaptations in fine root production and traits will alter water uptake capacity. In a managed C_3_ grassland, we tested the individual and combined effects of warming (+3°C), elevated CO_2_ (eCO_2_; +300 ppm) and drought on root water uptake (RWU) as well as on fine root production, trait adaptation, and fine root‐to‐shoot production ratios, and their relationships with RWU capacity. High temperatures, amplified by warming, exacerbated RWU reductions under drought, with negligible water‐sparing effects from eCO_2_. Drought, both under current and future (warming, eCO_2_) climatic conditions, shifted RWU towards deeper soil layers. Overall, RWU capacity related positively to fine root production and specific root length (SRL), and negatively to mean root diameters. Warming effects on traits (reduced SRL, increased diameter) and the ratio of fine root‐to‐shoot production (increased) were offset by eCO_2_. We conclude that under warmer future conditions, irrespective of shifts in water sourcing, it is particularly hot droughts that will lead to increasingly severe restrictions of grassland water dynamics.

## Summary statement

High temperatures, amplified by warming, exacerbate grassland water uptake reductions under drought, with negligible water‐sparing effects from eCO_2_ and drought shifting water sourcing to deeper soil layers. Root water uptake capacity is broadly related to fine root production and traits.

## 1 Introduction

Climate warming, rising atmospheric CO_2_ and severe drought are expected to modify the global water cycle (IPCC 2023; Naumann et al. 2018; Samaniego et al. 2020, 2018), increasing water limitation in many regions worldwide (Denissen et al. 2022; Grünzweig et al. 2022; Mankin et al. 2019; Teuling et al. 2010). These changes will affect the water dynamics in grasslands, which provide important ecosystem services and a significant agricultural resource (Schils et al. 2022), but are sensitive to water shortage (Fu, Ciais, Feldman, et al. 2022; Umair, Kim, and Choi 2020). In contrast to trees, grassland vegetation cannot store significant amounts of water, so its root water uptake (RWU) is closely coupled to transpiration in time (Aston and Lawlor 1979; Bakhshandeh et al. 2016). In C_3_ grassland, which dominates cool‐season regions (Havrilla et al. 2023; Winslow, Hunt, and Piper 2003), transpiration composes 85–95% of growing‐season evapotranspiration (ET) (Hu et al. 2014; Ma et al. 2020; Sun et al. 2021; Wang et al. 2015). How plants manage their transpiration (and thus, RWU) under warming, elevated CO_2_ (eCO_2_) and drought will, therefore, strongly determine the water dynamics of C_3_ grasslands in the future.

Transpiration in grasslands has been suggested to respond to warming, eCO_2_ and drought, each of which distinctly modifies water supply or demand. Warming increases ET in non‐water‐limited conditions by elevating the atmospheric water demand (vapour pressure deficit; VPD) (Grossiord et al. 2020; Sadok, Lopez, and Smith 2021). As conditions become drier, plants reduce stomatal conductance to avoid excessive water loss (Grossiord et al. 2020; Liang et al. 2023). Stomatal conductance is often downregulated under eCO_2_, which reduces water losses associated with photosynthetic carbon uptake and conserves soil moisture (soil water content; SWC) (De Kauwe, Medlyn, and Tissue 2021; Hatfield and Dold 2019; Liu et al. 2023; Morgan et al. 2011; Roy et al. 2016). Additionally, eCO_2_ can lead to increased biomass and transpiring leaf area, which could counterbalance potential water savings, although the magnitude of this effect is highly variable and remains poorly constrained (De Kauwe, Medlyn, and Tissue 2021; Walker et al. 2021). The effects of drought, warming and eCO_2_ on grassland transpiration (and thus, RWU), therefore, are relatively well understood individually. However, though future conditions are projected to involve changes of these global change factors in combination, their interactive effects are still unclear, particularly for C_3_ grasslands. Uncertainties also persist regarding whether and how these factors affect water sourcing within the rooting horizon. While a common assumption is that plant communities respond to drought by sourcing water from deeper soil layers, conflicting findings exist for grasslands. Some studies report shifts of RWU to deeper layers (Fischer et al. 2019; Guderle et al. 2018; Nippert and Knapp 2007; Weides et al. 2024; Wu et al. 2018), whereas others observed no change in water sourcing under drought (Deseano Diaz et al. 2023; Hoekstra et al. 2014; Prechsl et al. 2015). The effects of warming and eCO_2_ on grassland water sourcing (individually or combined) have not yet been studied, although both factors were found to interact with soil water potential across the rooting horizon (Forstner et al. 2023), suggesting they may alter water uptake patterns. Whether and how warming and eCO_2_ interact with potential drought effects on grassland water sourcing, however, remains unknown.

Fine root adaptation plays a key role in modulating water uptake under global change (Abdalla et al. 2022; Aroca, Porcel, and Ruiz‐Lozano 2012; Laughlin et al. 2023; Maurel and Nacry 2020). Grasslands allocate more of their biomass to fine roots when belowground resources (water, nutrients) are limited (Song et al. 2019; Zhou et al. 2020). Thus, root production in deep, wetter soil layers is often increased under drought (Bristiel et al. 2019; Comas et al. 2013; Li et al. 2022). Further possible adaptations to water shortage include trait changes, such as increased specific root length (SRL; root length per unit mass) and decreased root diameters (Cusack et al. 2024; Fort et al. 2017; Freschet et al. 2021; Reich, Hobbie, and Lee 2014; Roumet et al. 2016). Rather than maximizing resource uptake by individual roots (Kong et al. 2019; Zhang et al. 2023), higher SRL and reduced diameters can enhance community‐level resource uptake by increasing the soil volume explored per unit biomass (Cusack et al. 2024; Freschet et al. 2021) and are thus considered to represent a ‘resource‐acquisitive’ strategy. In general, climate warming tends to promote such trait adaptation amongst fine roots (Björk et al. 2007; Carrillo et al. 2014; Chandregowda et al. 2023; Nelson et al. 2017; Wang et al. 2021). Furthermore, as water availability decreases, warming commonly stimulates belowground biomass allocation, increasing root‐to‐shoot ratios (Song et al. 2019; Zhou et al. 2020). In wetter conditions, however, warming may instead reduce biomass allocation to roots (Song et al. 2019). The fate of fine root production under warming, similarly, varies throughout the literature and is context‐dependent (Wang et al. 2021), creating uncertainty about the effects in C_3_ grassland. In contrast, eCO_2_ typically increases plant nutrient demand, and therefore frequently promotes belowground biomass allocation (Zhou et al. 2020). Concurrently, finer shallower root systems tend to develop, which may increase near‐surface nutrient acquisition (Arnone et al. 2000; Mueller et al. 2018; Song et al. 2019). These eCO_2_ responses, however, can change altogether under experimental warming (Arndal et al. 2018; Carrillo et al. 2014) and should have implications for RWU during drought (Wang, Wang, and Liu 2022). To date, few studies have investigated how fine roots respond to the individual versus combined treatments of warming, eCO_2_ and drought across different soil layers in a grassland (Arndal et al. 2018; Carrillo et al. 2014; Mueller et al. 2018), with no consideration of C_3_ grasslands. Therefore, it remains largely unknown how these global change factors, individually and combined, affect the production, diameter and SRL of fine roots, and how these changes across the soil profile alter the capacity of the grassland to take up water.

This study addressed these knowledge gaps using a unique multifactor global‐change experiment in a managed C_3_ grassland in central Austria, where grassland was exposed to individual and combined treatments of warming (+3°C), eCO_2_ (+300 ppm CO_2_) and drought, as broadly expected under “business as usual” activity by 2100 (IPCC 2023). Our question was twofold: first, we asked how these three global change factors individually and interactively affect grassland RWU across the different layers of the main rooting horizon. Second, we sought to identify the effects of these factors on the production of fine roots as well as their diameter and SRL, and the ratio of fine root‐to‐shoot production. We further aimed to establish how the mass, diameter and SRL of newly produced fine roots related to RWU across global change treatments. For the first part, we hypothesized that (i) warming would increase RWU rates, eCO_2_ would decrease RWU and partly offset warming effects, drought would reduce RWU while shifting water sourcing to deeper soil layers, and that future conditions (warming combined with eCO_2_) would amplify the effects of drought. For the second part, we hypothesized that (ii) individual treatments would increase the ratio of fine root‐to‐shoot production and promote thinner diameters and higher SRL, with root production increasing predominantly in topsoil layers under eCO_2_, across the rooting horizon under warming, and in deeper soil layers under drought, while combined treatment effects would be additive. We furthermore expected that (iii) across treatments, the capacity of grassland to take up water would positively relate to fine root production and SRL, and negatively relate to root diameters. Addressing these hypotheses revealed how warming, eCO_2_ and drought individually and interactively affected RWU as well as the production and key traits of fine roots and biomass allocation in this managed C_3_ grassland, defining its ecohydrological response to projected future conditions.

## 2 Materials and Methods

### 2.1 Study Site

This study used a long‐term multifactor global‐change experiment (‘ClimGrass’) in a permanent managed C_3_ grassland located at the AREC Raumberg‐Gumpenstein research facility near the central European Alps in Styria, Austria (47°29′44.6″N, 14°5′54.6″E) (Maxwell et al. 2022; Reinthaler et al. 2021). This site, which is located at 695 m a.s.l., receives 1077 mm annual precipitation and has a mean annual temperature of 8.5°C, is typical for lower montane valleys of the Alps. The soil is classified as Dystric Cambisol (arenic, humic; IUSS Working Group WRB 2015) with a loamy sand texture, with a sand content of 44.2%, silt content of 47.6%, clay content of 8.3%, a C:N ratio of 12.6:1, and a pH‐value of ~5.5 (Reinthaler et al. 2021). The dominant plant species include C_3_ grasses (*Arrhenatherum elatius* L., *Poa pratensis* L., *Festuca pratensis* L., *Dactylis glomerata* L.) and forbs (*Taraxacum officinalis*, *Trifolium repens*, *Plantago lanceolata*). Grasses make up about 84% of the canopy biomass, while leguminous and nonleguminous forbs contribute 14% and 2%, respectively (Joseph et al. 2025). The grassland management practice consists of three cuts per year, whose timing follows the phenological development and thus the local traditional practice (late May, late July, and early October). After each cut, nutrient removal through harvest is compensated by fertilization with nitrogen, phosphorous and potassium in amounts totalling 90, 28 and 140 kg ha^−1^ per year, respectively. This study took place during the growing seasons (April until October) of 2017, 2019, and 2020.

### 2.2 Experimental Design

This study used 25 plots (4 × 4 m) exposed to six global change treatments: (i) ambient conditions (control; *n* = 8); (ii) drought (*n* = 4); (iii) warming (*n* = 3); (iv) eCO_2_ (*n* = 3), (v) future conditions (warming and eCO_2_; *n* = 3); and (vi) drought under future conditions (warming, eCO_2_ and drought; *n* = 4). The applications of warming and eCO_2_ started in 2014 and use equipment mounted on aluminium frames, suspended over plots and raised with canopy growth, always applying these treatments at the canopy surface. Warming was achieved using six 500‐W infrared heaters, which aimed to increase canopy surface temperatures by 3°C except when snow cover exceeded 10 cm. The warming treatment varied negligibly over time, and similarly positioned sensors and ingrowth cores across plots accounted for a slight thermal gradient (Pötsch et al. 2020). For the eCO_2_ treatment, a mini free‐air CO_2_ enrichment (FACE) system (Miglietta, 2001) aimed to raise CO_2_ concentrations by 300 ppm by fumigating grassland during daytime hours (global radiation > 50 W/m²) throughout growing seasons, generally staying within ± 70 ppm of the target with any variations mainly being due to wind (Pötsch et al. 2020). In several ambient and future‐condition plots, automatic shelters which completely exclude precipitation are activated during summer to induce droughts (23/05–27/07 in 2017, 18/04–17/06 in 2019, and 17/06–29/07 in 2020), ending with a cut and a rewetting with 40 mm of collected rainwater (Reinthaler et al. 2021). These treatments permit a comparison of drought effects under current versus future conditions and, additionally, give insight into the individual and combined effects of climate warming and eCO_2_.

### 2.3 Environmental Data

SWC measurements were collected at four depths (3, 9, 18, and 36 cm; 15‐min resolution) in two plots per treatment using time‐domain reflectometry sensors (Delta‐T SM150, METER Group, Munich, Germany) connected to a Datalogger (CR1000, Campbell Scientific, Logan, UT, USA). These SWC time‐series were cleaned by removing values outside the 0–0.6 cm^3^ cm^−3^ range, smoothing trends using hourly medians, and gap‐filling 2.9% of the data. Spike detection was then applied to remove final single time‐step outliers (Dorigo et al. 2013). Canopy‐height air temperature and relative humidity—used to calculate VPD—were recorded per treatment using sensors (CS215‐L, Campbell Scientific, Logan, UT, USA). An on‐site weather station belonging to GeoSphere Austria (http://www.geosphere.ac.at) recorded rainfall.

### 2.4 Root Water Uptake Estimation

Diurnal fluctuations in SWC were used to derive RWU estimates for the main rooting horizon of this grassland using an approach described by Guderle and Hildebrandt (2015; see multi‐step, multi‐layer regression). For an extended methodology and concept figure, see Supporting Information S1. This approach attributes daytime SWC declines (*∂*SWC*/∂t*_day_) during non‐rain periods to a combination of ET and subsequent vertical flow that fills the potential energy gap. Vertical flow persists overnight, but as solar energy decreases, plants close their stomata and atmospheric evaporative demand is low, thus transpiration rates approach zero (Loheide, 2008). Assuming that (1) ET is negligible at night and (2) vertical flow is similar at day and night, this approach considers night‐time SWC changes during non‐rain periods (*∂*SWC*/∂t*_night_) to be solely derived from vertical flow. These assumptions are particularly sound during non‐rain periods (Groh et al. 2019) and have been validated as sufficiently accurate to obtain robust RWU estimates (Guderle and Hildebrandt 2015; Hupet et al. 2002; Li et al. 2002). Previous work has shown that, by fitting linear models to separate day and night segments of SWC cycles and adjusting the daytime slopes based on the night‐time slopes, ET can be effectively isolated (Chai et al. 2023; Jackisch et al. 2020; Lai et al. 2023; Li et al. 2002; Renner et al. 2016). As transpiration dominates the growing‐season ET in temperate C_3_ grasslands—that is, its contributions approach 100% between rainfall events (Dubbert et al. 2013; Hu et al. 2014; Ma et al. 2020; Sun et al. 2021; Wang et al. 2015), and we excluded periods with potentially reduced transpirational contributions from statistical analyses (i.e., the weeks during which the grassland canopy was developing following snow disappearance, and 2 weeks following grassland cuts), we consider the soil‐moisture changes isolated here to be RWU, associated with transpiration. RWU estimates were calculated across the soil profile (3, 9, 18 and 36 cm) using:

$$\mathrm{RWU}=t_{\mathrm{day}}\left(\frac{\partial SWC}{\partial t_{\mathrm{day}}}+\frac{\partial SWC}{\partial t_{\mathrm{night}}}\right)$$

where *t*_day_ is the number of daytime hours, and *∂*SWC*/∂t*_day_ and *∂*SWC*/∂t*_night_ each require ≥ 4 data points. Day and night were defined using standard Austrian sunrise and sunset times, and estimates were accepted for days with negligible precipitation (< 2 mm). Per‐depth estimates were then integrated into a depth‐weighted average for the main rooting horizon (0–36 cm, using the 3 cm estimate for the 0–3 cm zone) to get RWU in mm day^−1^.

We also examined how RWU varies according to the hourly magnitudes of SWC and VPD, which can be highly dynamic (Zhou et al. 2014). To achieve this, we replaced the linear *∂*SWC*/∂t*_day_ in the above equation with a polynomial model of daytime SWC to obtain hourly RWU (see Supporting Information S1 for concept figure):

$$f\left(t_{\mathrm{day}}\right)=at_{\mathrm{day}}+bt_{\mathrm{day}}^{2}+ct_{\mathrm{day}}^{3}$$

where *a*, *b* and *c* are polynomial coefficients and ≥ 8 data points are required. Slopes from the polynomial SWC models were extracted at each daytime hour (*∂f(t*_day,h_)) and adjusted based on the linear nighttime trend (*∂SWC/∂t*_night_), like for daily RWU:

$${\mathrm{RWU}}_{h}=\partial f\left(t_{\mathrm{day},h}\right)+\frac{\partial SWC}{\partial t_{\mathrm{night}}}$$

where *h* is a daytime hour between 1 and *t*_day_. Like for daily estimates, these hourly per‐depth RWU estimates were integrated across the main rooting horizon to obtain RWU in mm h^−1^.

To evaluate how SWC and VPD interactively affected these hourly RWU estimates, data were aggregated into SWC and VPD deciles which were analysed independently (Bachofen et al. 2023; Fu, Ciais & Prentice 2022; Yu et al. 2022). RWU responses to SWC and VPD were then compared between treatments from two perspectives: across VPD deciles, we compared *RWU*_*SWC*_ (RWU/SWC), indicating the fraction of SWC taken up by roots. Conversely, across SWC deciles, we compared *RWU*_*VPD*_ (RWU/VPD), indicating RWU relative to VPD and reflecting trends in canopy conductance, typically calculated using eddy‐covariance measurements of the latent heat flux (Arneth et al. 1996; Köstner et al. 1992; Pasqualotto et al. 2021). This approach revealed how global change treatments altered RWU over varying soil and atmospheric water availability.

### 2.5 Fine Roots and Aboveground Biomass

Fine roots (diameter ≤ 1 mm) produced during each of the three years were sampled using ingrowth cores (4 cm diameter, 30 cm deep, 6 mm mesh size) (Maxwell et al. 2022; Roy et al. 2016). Compared to methods measuring standing root mass and traits, this approach minimizes plot destruction, which is crucial to maintain this long‐term experiment. Data obtained provide insight into long‐term fine root production dynamics and permit comparison of the portion of the root system most active in water and nutrient uptake amongst global change treatments (Li et al. 2013). Cores were extracted from each plot at the aboveground cuts performed three times per year and split into segments representing three soil layers (0–10, 10–20, and 20–30 cm). Cores were re‐inserted afterwards (filled with fresh, sieved soil sourced from the site) to measure the fine root growth until the next aboveground cut. Vegetation samples, taken from 1 m^2^ in each plot, were collected to determine how the production of fine roots compared to that of the aboveground (i.e., ‘shoot’) biomass. All samples were stored at −18°C. Upon thawing, core samples were sieved to obtain any fine roots, which were scanned for their total length, projected area, and mean diameter (WinRhizo software). All samples (i.e., scanned roots and thawed vegetation) were oven‐dried at 60°C for 4 days. Afterwards, samples were weighed to determine the fine root and aboveground biomass produced during periods between grassland cuts. We then calculated the per‐layer SRL,

$$\mathrm{SRL}=\frac{\mathrm{total}\;\mathrm{fine}\;\mathrm{root}\;\mathrm{length}\;\left(m\right)}{\mathrm{fine}\;\mathrm{root}\;\mathrm{dry}\;\mathrm{mass}\;\left(g\right)}$$

specific root area (SRA),

$$\mathrm{SRA}=\frac{\mathrm{projected}\;\mathrm{fine}\;\mathrm{root}\;\mathrm{area}\;\left(m^{2}\right)}{\mathrm{fine}\;\mathrm{root}\;\mathrm{dry}\;\mathrm{mass}\;\left(g\right)}$$

and the ratio of fine root‐to‐shoot production for the grassland as

$$R/S\;\mathrm{production}=\frac{\mathrm{fine}\;\mathrm{root}\;\mathrm{dry}\;\mathrm{mass}\;\left(0–30\;\mathrm{cm}\right)}{\mathrm{aboveground}\;\mathrm{dry}\;\mathrm{biomass}}$$

To understand how the mass of fine roots per unit volume of soil, i.e., fine root mass density (mg cm^−3^), and their traits at each sampling affected the capacity of grassland to take up water (RWU capacity) across the soil profile, we analysed the relationships of these properties with the maximum hourly water uptake (*RWU*_*max*_) per soil layer in each plot. *RWU*_*max*_ for each soil layer was derived from interpolated RWU profiles across depth over the last 10 days before root extraction, typically including a rainfall event to allow RWU to approach capacity. We then compared how these root properties influenced *RWU*_*max*_ across different treatments, considering potential treatment effects.

### 2.6 Statistical Analyses

To analyse the effects of global change treatments on response variables, we employed linear mixed‐effects models using the ‘lmer’ function in R (*lme4* package; Bates et al. 2015; Bolker 2024). For an extended methodology, see Supporting Information S2. The model was formulated as ‘lmer (response variable ∼ treatment × other + (1|time) + (1|space), data = data)’. The response variables in our models were total daily RWU, fractions of RWU across soil layers in the main rooting horizon, *RWU*_*SWC*_ and *RWU*_*VPD*_, fine root production and studied traits, the ratio of fine root‐to‐shoot production, and *RWU*_*max*_. Fixed effects were global change treatments and any other parameter(s) required to further constrain these treatment effects (indicated in the model formula by ‘other’), as well as their interaction. Examples of these ‘other’ fixed effects include (1) the year and period of the growing season, which were often required to assess drought effects during versus outside rainfall exclusion, and (2) fine root properties (the mass density of newly produced roots, and the studied root traits), for which we wanted to know how they related to *RWU*_*max*_. Random effects controlled for variability to time (period of the growing season nested within years, or daily/hourly variation) and space (plots, grouped by location across the experiment to assess spatial balance), minimizing pseudoreplication (Metze et al. 2023). Across models, the variance of random spatial effects was small compared to residual variance (the former composing largely < 2% of the latter; Supporting Information S1: Tables S1‐6), indicating minimal spatial imbalance across the experiment, and that plots can be considered ‘true’ treatment replicates (Meeran et al. 2021). Random time effects ensured that any treatment effects were derived from consistent responses.

We used restricted maximum likelihood estimation to estimate the fixed and random effects coefficients in our models. To improving the balance between model complexity and fit, statistically insignificant interaction terms were removed where this lowered the Akaike information criterion. This only affected models where the response variable was *RWU*_*max*_ and predictor variables included fine root traits; the interaction terms in all other models were retained in the final model specifications (S2). Model fits were assessed using the marginal *R* ^*2*^ (*R²m*, the proportion of variance explained by fixed effects) and conditional *R* ^*2*^ (*R²c*, the proportion of variance explained by fixed and random effects) (Nakagawa and Schielzeth 2013), derived using the function ‘r.squaredGLMM’ (*MuMIn* package; Bartoń 2024).

To compare data distributions amongst treatments, we used the ‘emmeans’ function (*emmeans* package; Lenth 2024) and compared distributions with the ‘cld’ function (*multcomp* package; Hothorn 2023) using *α* = 0.05 and false discovery rate correction for multiple testing (e.g., six treatments). To meet the assumptions of normality in linear mixed effects modelling, we confirmed the normality of model residuals using Q–Q plots. Residuals plotted against fitted values showed no clear patterns, indicating reliable coefficient estimation (homoscedasticity, no outliers). All statistical analyses were conducted using R software version 4.3.2 (R Core Team 2023).

## 3 Results

### 3.1 Grassland Water Uptake under Individual and Combined Global Change

**Figure 1:**
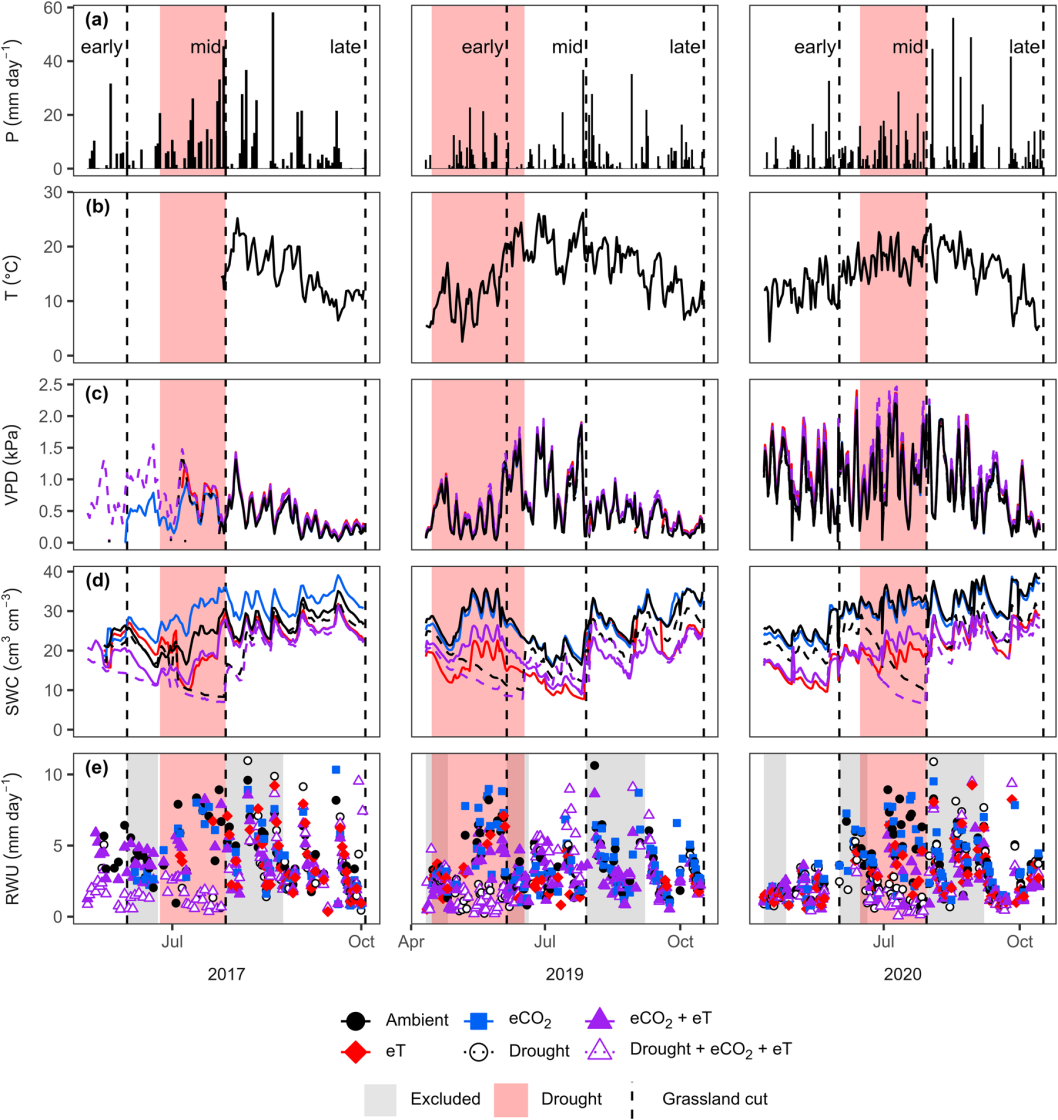
(a) Daily precipitation (P) and (b) mean values of air temperature (T), (c) vapour pressure deficit (VPD), (d) soil water content (SWC), and (e) root water uptake (RWU) in a grassland exposed to individual and combined treatments of warming (eT; +3°C in canopy surface temperatures), elevated CO_2_ (eCO_2_; +300 ppm), and drought. RWU analyses focused on timeframes excluding periods following grassland cuts or snow cover (see methods). Growing season periods, indicated in the top panel, are separated by grassland cuts. For individual displays of all 12 plots, see Figure S2.

Global change treatments had a significant effect on daily grassland RWU, larger than that of annual variation or the period of the growing season (*p* < 0.001; see Table S1 for detailed statistics, including *F* values). Treatment effects were most pronounced during mid‐seasons (periods between the first and the second cut; Figure 1a), which were hot and dry in 2019 (Figure 1a,b) and involved exceptionally high VPD in 2020 (Figure 1c). At these times, VPD in treatments involving warming was particularly high (Figure S3) while SWC was reduced (Figure S4). The experimental droughts resulted in a pronounced SWC decrease (Figures 1d and S4).

**Figure 2:**
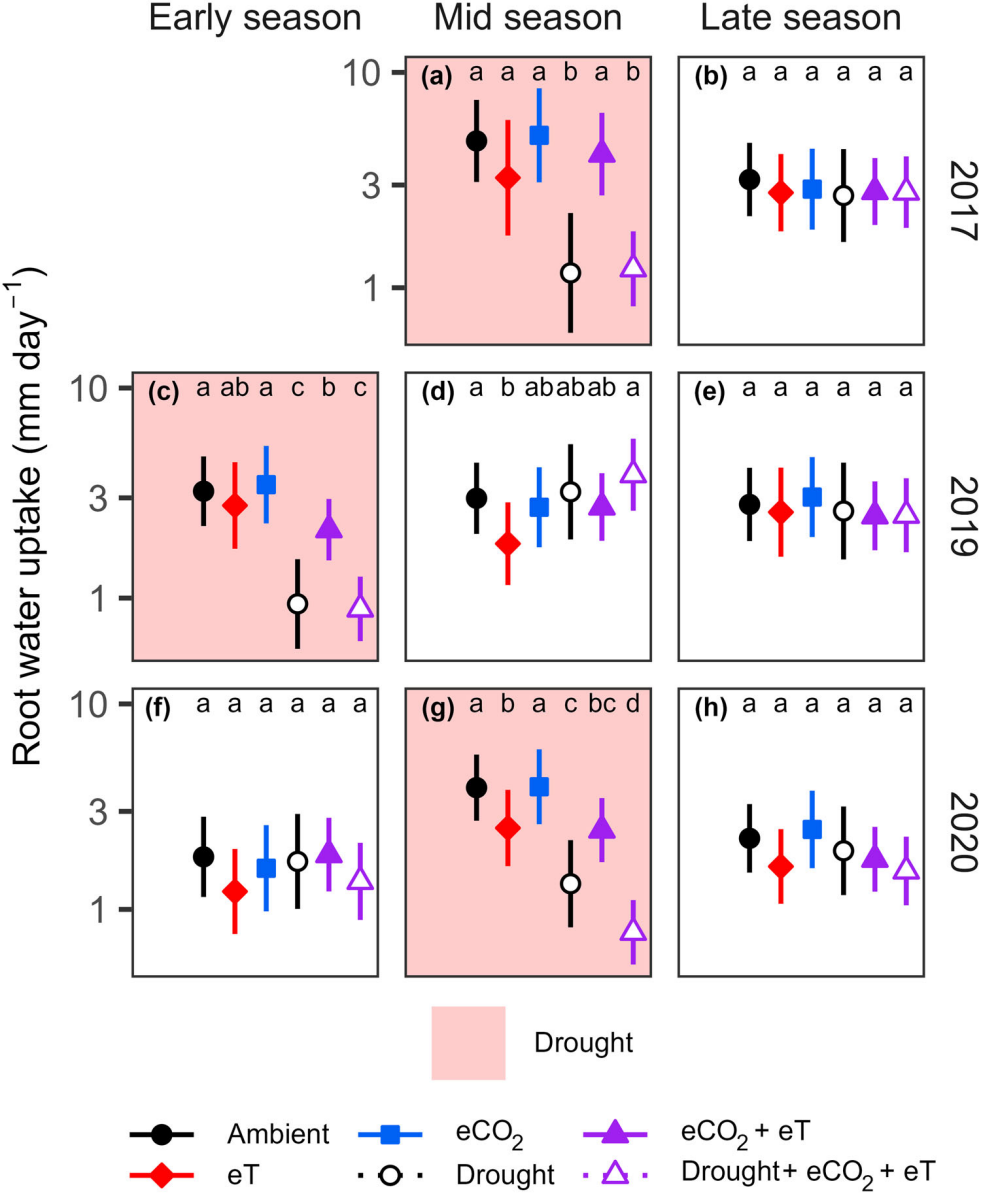
Mean daily root water uptake in a grassland exposed to individual and combined treatments of warming (eT; +3°C), elevated CO_2_ (eCO_2_; +300 ppm), and drought. For the detailed statistics, see Table S1. Letters compare daily root water uptake between treatments during periods of the three growing seasons (a–h); treatments not sharing any letter differ in daily uptake at the 5% significance level. Error bars show 95% confidence intervals. [Color figure can be viewed at wileyonlinelibrary.com]

During mid‐seasons over the study period, when temperatures peaked (Figure 1b), warming frequently reduced daily RWU (by 29–40%; Figure 2d,g; *p* < 0.05; see Table S1 for the detailed statistics). In contrast, no significant change in these RWU values occurred under eCO_2_ at any time. Future conditions reduced daily RWU to a similar magnitude as warming alone (by ~35%; Figure 2c,g; *p* < 0.01) though eCO_2_ tended to offset the warming effect during the 2019 mid‐season, which was the hottest period (Figure 2d; *p* = 0.09). Experimental droughts reduced daily RWU similarly in ambient and future conditions in 2017 and 2019 (by 70–76%; Figure 2a,c; *p* < 0.0001). Yet during the 2020 drought, which coincided with comparatively high temperatures and VPD (Figure 1b,c), RWU reductions under future conditions were amplified by 20% (Figure 2g; *p* < 0.05). Overall, the model explained significant variance in daily grassland RWU using treatment effects during specific periods (Table S1; *R²m* = 0.34) and explained most of this variance when daily variability was considered (*R²c* = 0.86).

**Figure 3:**
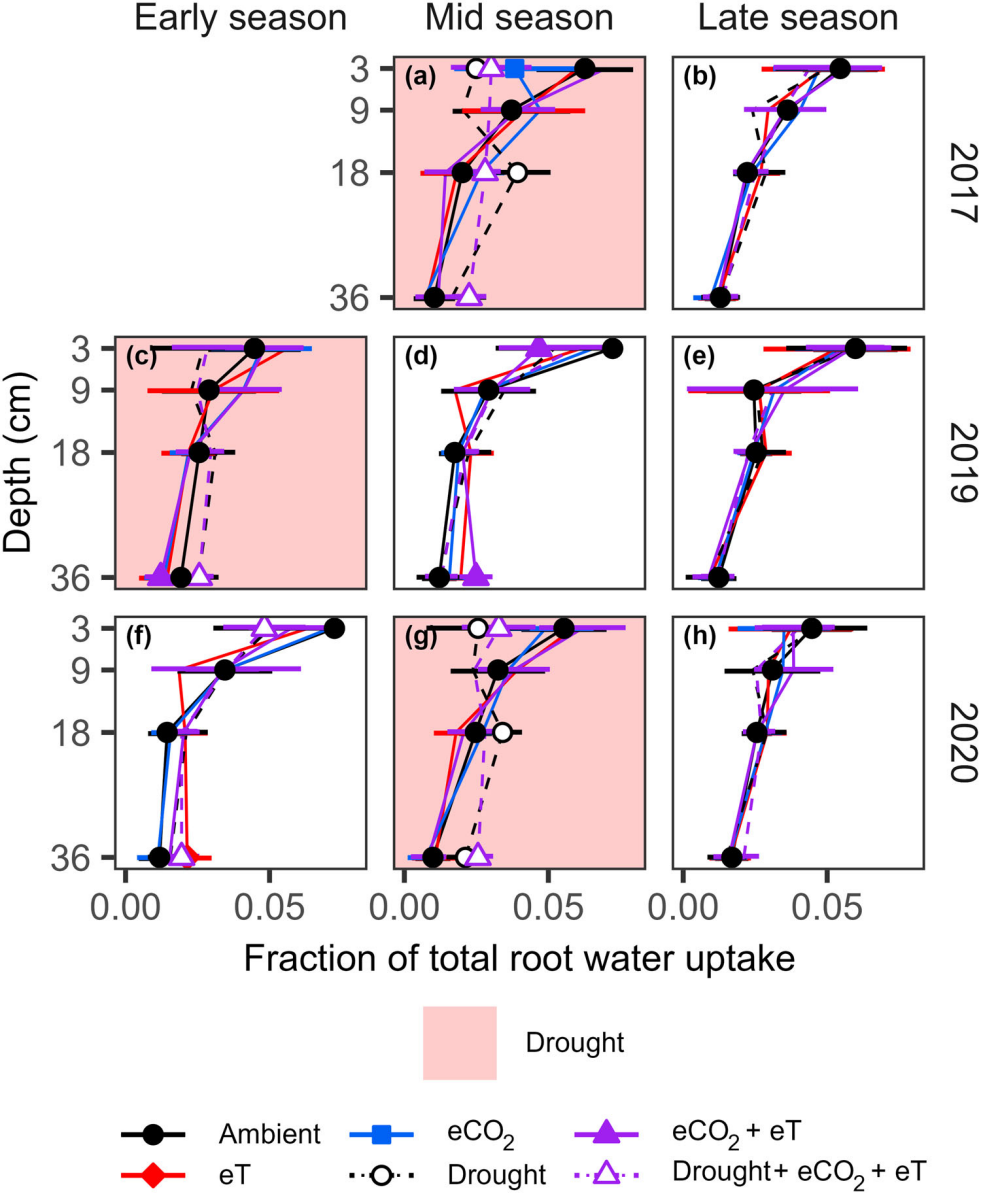
Fractions of total root water uptake in the main rooting horizon of a grassland under individual and combined treatments of warming (eT; +3°C), elevated CO_2_ (eCO_2_; +300 ppm) and drought, compared per depth during periods of three growing seasons (a–h). For the detailed statistics, see Table S2. Treatment symbols, where present, indicate differences from ambient levels at the 5% significance level. Error bars show 95% confidence intervals. [Color figure can be viewed at wileyonlinelibrary.com]

The portion of variance in the fractions of total RWU explained by treatment effects over time increased towards deeper soil layers (Table S2; indicated by the increase in *R²m* from 0.16 to 0.36 with depth). Specifically, the droughts increased water sourcing (i.e., fractions of total water uptake) in the deepest soil layer, regardless of ambient or future conditions (Figure 3a,c,g). The magnitude of this increase varied between 20% and 50% (*p* < 0.05; see Table S2 for the detailed statistics) and meant that RWU during drought was more uniformly distributed throughout the rooting horizon. Following experimental droughts, grassland resumed a sourcing of water which more closely resembled that of the ambient treatment. In contrast to drought, warming and eCO_2_—individually or combined—had no consistent significant effects on grassland water sourcing.

**Figure 4:**
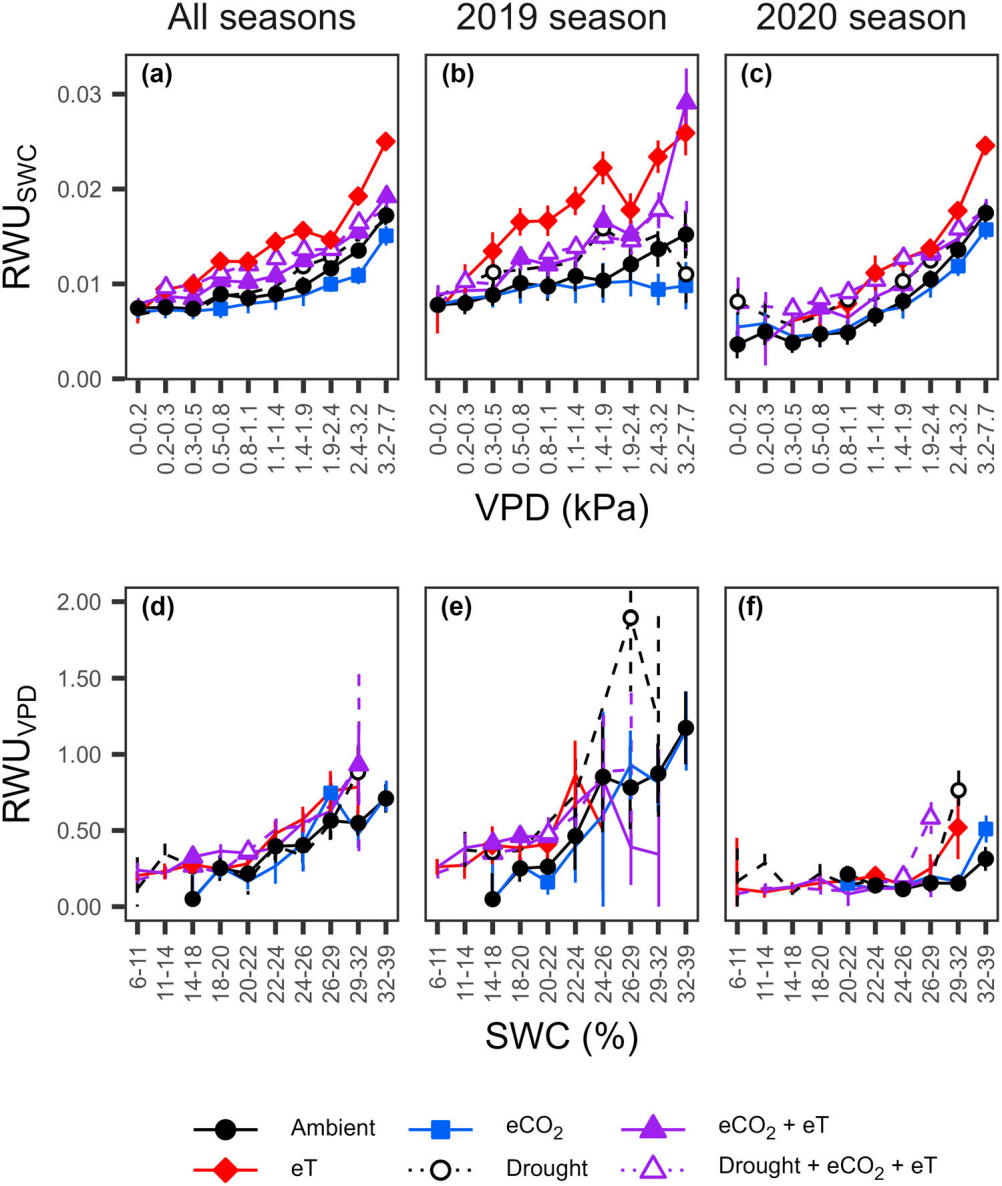
Effects of vapour pressure deficit (VPD) and soil water content (SWC) on hourly root water uptake (RWU) in a grassland exposed to individual and combined treatments of warming (eT; +3°C), elevated CO_2_ (eCO_2_; +300 ppm), and drought. The top panels show (a) *RWU*_*SWC*_ (RWU/SWC, indicating the fraction of SWC taken up by roots) across VPD deciles during all growing seasons and individual seasons of (b) 2019 and (c) 2020. The bottom panels show *RWU*_*VPD*_ (RWU/VPD, indicating the extent of RWU relative to VPD, reflecting canopy conductance) across SWC deciles for the respective periods (d–f). For the detailed statistics for *RWU*_*SWC*_ and *RWU*_*VPD*_, see Tables S3 and S4, respectively. Treatment symbols, where present, indicate differences from ambient levels at the 5% significance level. Error bars show 95% confidence intervals. [Color figure can be viewed at wileyonlinelibrary.com]

Generally, RWU increased with SWC and VPD; however, effects of SWC and VPD on RWU interacted differently between treatments (Figure S5a,b). This was reflected by how treatments altered VPD effects on *RWU*_*SWC*_ (indicating the fraction of SWC taken up by roots), whereas treatments did not strongly influence how SWC affected *RWU*_*VPD*_ (RWU relative to VPD, reflecting trends in canopy conductance). Treatment effects on *RWU*_*SWC*_ intensified with increasing VPD (Figure 4a), indicated by minimal *RWU*_*SWC*_ variance for lower VPD deciles but up to 20–25% of variance in the upper deciles (see *R²m* in Table S3). As VPD increased, *RWU*_*SWC*_ was enhanced under warming (*p* < 0.01; see Table S3 for the detailed statistics) and reduced under eCO_2_ beyond a VPD threshold of 1.9 kPa (*p* < 0.05). Under warming, eCO_2_ reduced *RWU*_*SWC*_ over a broader range of VPD (> 0.2 kPa vs. > 1.9 kPa; *p* < 0.05), approximately halving the warming‐induced increase in *RWU*_*SWC*._ Drought increased *RWU*_*SWC*_, although this was statistically evident only within the VPD range of 1.4–2.4 kPa (*p* < 0.05), and heightened *RWU*_*SWC*_ under future conditions (*p* < 0.05). *RWU*_*VPD*_ increased with SWC deciles but, in contrast to *RWU*_*SWC*_, showed inconsistent responses to global change treatments (Figure 4d), which only explained negligible proportions of variation (see *R²m* and the detailed statistics in Table S4). Both *RWU*_*SWC*_ and *RWU*_*VPD*_ exhibited annual variation. During 2020, *RWU*_*VPD*_ was comparatively low and insensitive to SWC (Figure 4f), while *RWU*_*SWC*_ and its treatment sensitivity were reduced (Figure 4c). Conversely, in 2019, *RWU*_*VPD*_ was comparatively high and sensitive to SWC (Figure 4e), while *RWU*_*SWC*_ and its treatments sensitivity were enhanced (Figure 4b).

**Figure 5:**
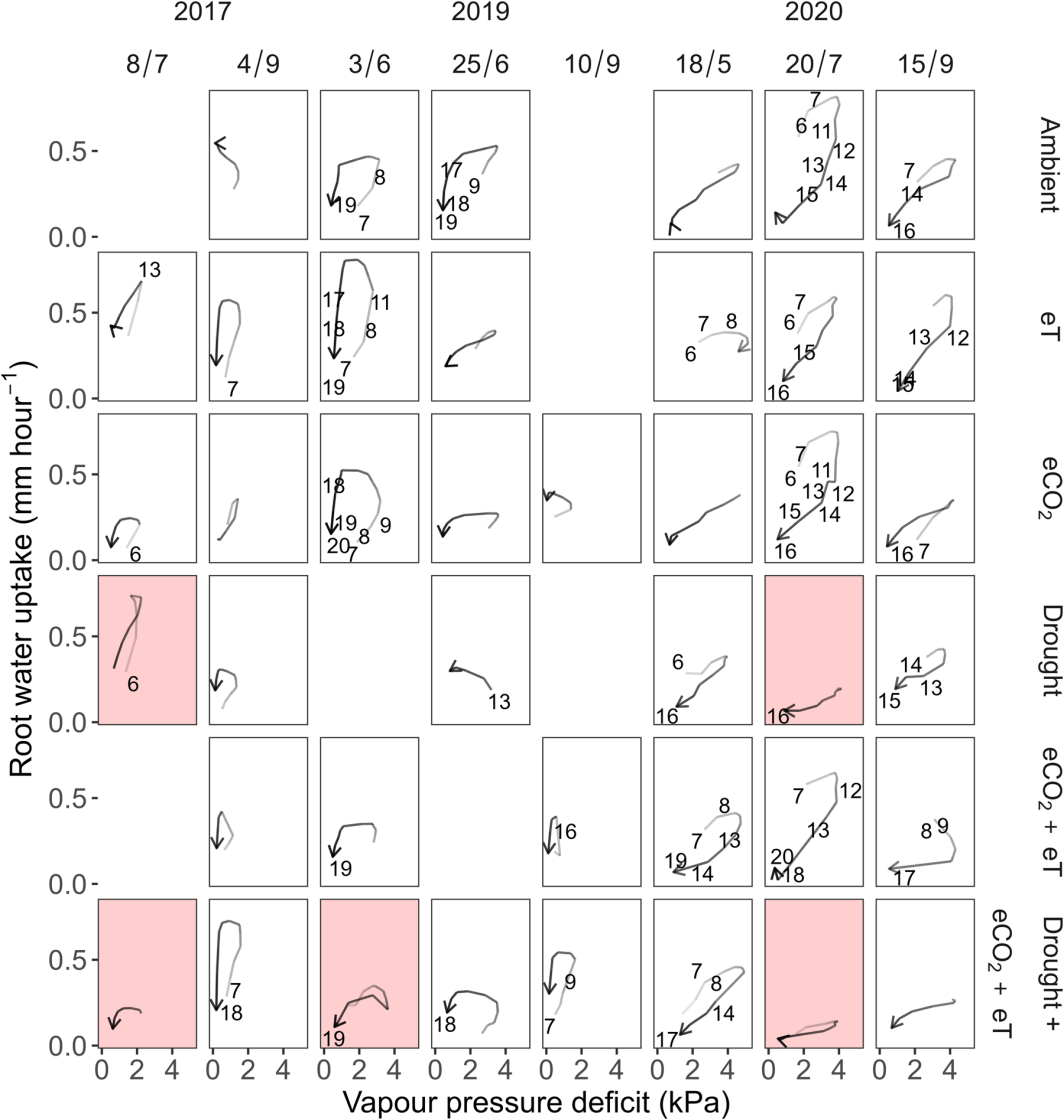
Diurnal relationships between vapour pressure deficit and hourly root water uptake in a grassland exposed to individual and combined treatments of warming (eT; +3°C), elevated CO_2_ (eCO_2_; +300 ppm), and drought. Treatment plots and days (dd/m) with the most complete data records were selected for display. Numbers represent the hour of the day in 24h‐time. Red shading indicates drought periods. [Color figure can be viewed at wileyonlinelibrary.com]

The annual variability of RWU_VPD_ was underpinned by changing diel cycling between RWU with VPD, shown as a shifting hysteretic relationship (Figure 5). In 2017 and 2019, when VPD remained relatively low (Figures 1c and S4), RWU increased as VPD rose but exhibited a lagging decrease as VPD dropped (Figure 5). In 2020, daily VPD peaks increased, causing RWU to decline earlier relative to VPD peaks. During this time, drought almost eliminated this hysteresis.

### 3.2 Fine Roots under Global Change Treatments

**Figure 6:**
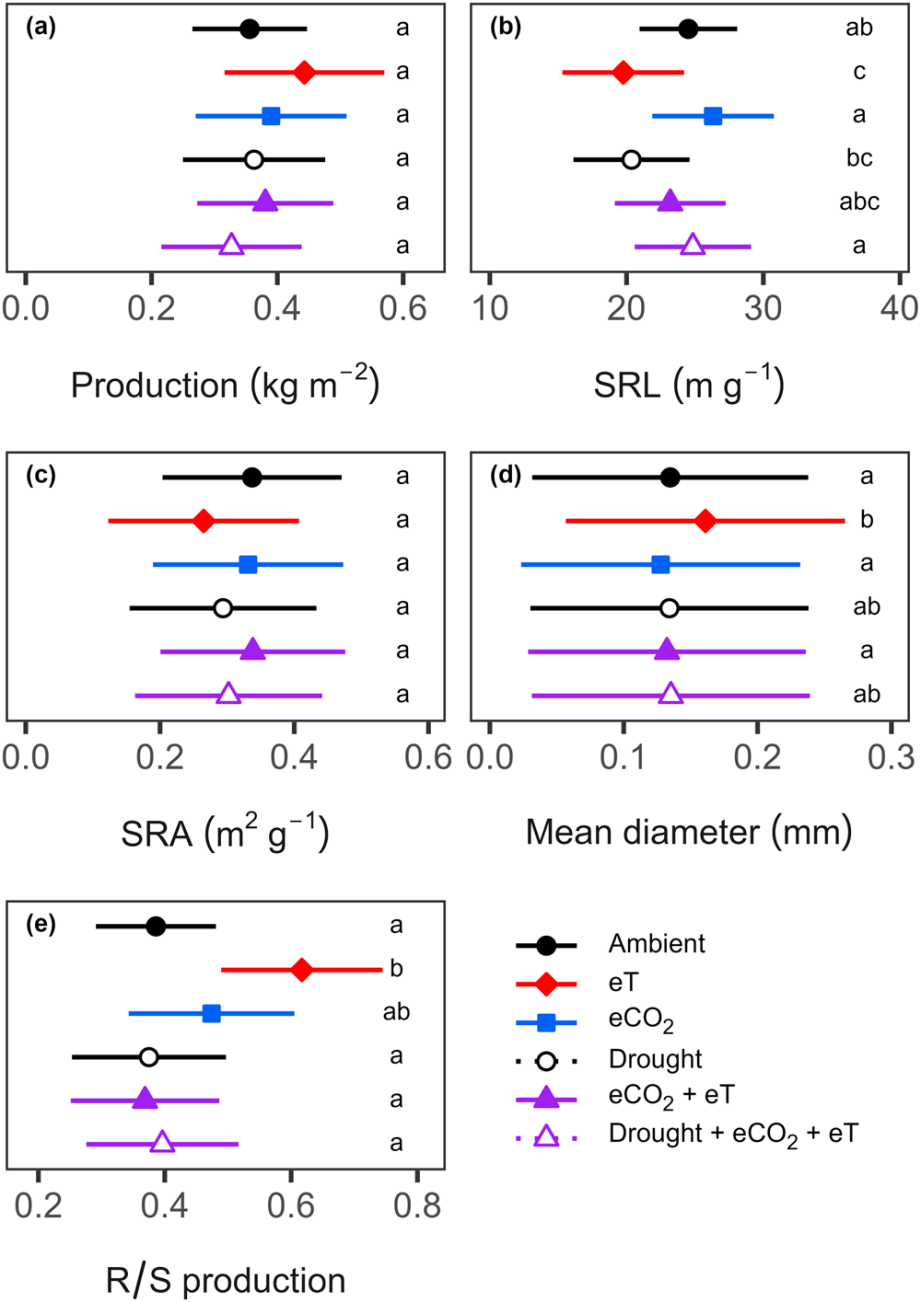
(a) Annual fine roots production (b), specific root length (SRL), (c) specific root area (SRA), (d) mean root diameters, and (e) the ratio of fine root‐to‐shoot production (R/S production) in a grassland exposed to individual and combined treatments of warming (eT; +3°C), elevated CO_2_ (eCO_2_; +300 ppm), and drought. Roots were extracted three times per growing season in 2017, 2019 and 2020. For the detailed statistics, see Table S5. To view data over time and soil layers, see Figures S6 and S7, respectively. Mean values not sharing any letter within facets differ at the 5% significance level. Error bars denote 95% confidence intervals. [Color figure can be viewed at wileyonlinelibrary.com]

Fine roots, sampled from the grassland main rooting horizon three times per growing season, adapted to sustained warming (Figure 6b,d,e and S6). While warming had no clear effect on fine root production, it increased the ratio of fine root‐to‐shoot production (by 48%; Table S5; *p* < 0.01). Furthermore, this treatment led to reduced SRL (by 20%; *p* < 0.05) and increased the mean diameters (by 19%; *p* < 0.01) of fine roots produced, particularly during 2020 (Figure S6). All these warming‐induced changes were offset by eCO_2_ (Figure 6b,d,e, Table S5). However, we found no significant evidence for increased fine root biomass allocation and changed topsoil (0–10 cm) rooting under eCO_2_ alone. Moreover, we found only a tendency towards less surface root production under drought in future conditions (Figure S7; *p* = 0.106). Thus, treatment effects explained relatively little of the variance in the mass and studied traits (diameter, SRL, SRA) of newly produced fine roots and the ratio of fine root‐to‐shoot production during this study overall (Table S5; *R²m* values < 0.1). The total length and surface area of fine roots produced, which we also examined, exhibited no treatment effects.

### 3.3 Fine Root Adaptations and Maximum Water Uptake

**Figure 7:**
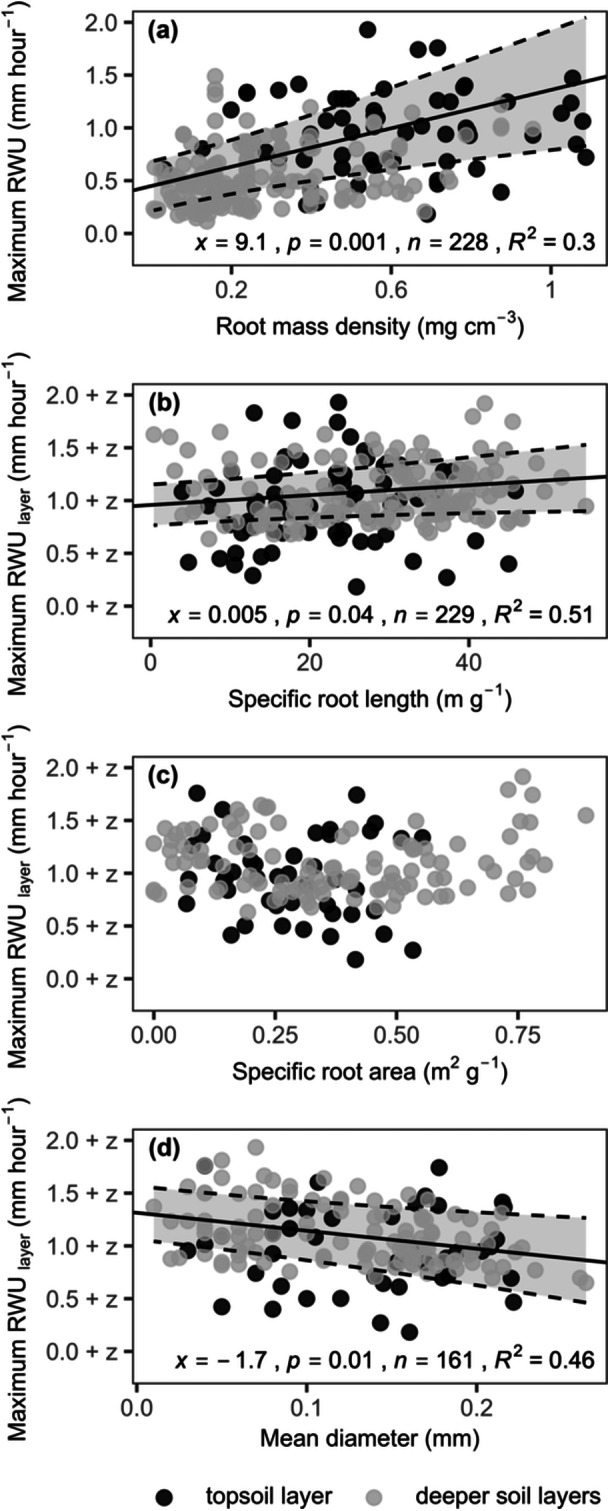
Relationships between the maximum hourly root water uptake (RWU) and (a) the root mass density, (b) specific root length, (c) specific root area, and (d) mean diameter of fine roots produced in topsoil (0–10 cm) and deeper soil layers (10–20, 20–30 cm) of a grassland. Trendlines indicate effects on maximum RWU across all layers at the 5% significance level. For traits (b–d), data from deeper soil layers are offset using depth coefficients (*z*; see model summaries in Table S6) to highlight the consistency of effects on maximum RWU across the profile. Labels report the slope (*x*), *p*‐value, sample number (*n*), and conditional *R* ^*2*^ value of the linear mixed‐effect models. Roots were extracted three times per growing season in 2017, 2019 and 2020 and matched to the maximum hourly RWU in the same place.

Over the study period, variations in fine root production and studied traits related to the capacity of grassland to take up water, as indicated by *RWU*_*max*_ (the maximum hourly RWU recorded earlier in the same plot). Fine root production positively influenced *RWU*_*max*_ (Figure 7a; *p* < 0.001; see Table S6 for the detailed statistics). Furthermore, across soil layers, the SRL of these roots related positively to *RWU*_*max*_ (Figure 7c, Table S6; *p* < 0.05) while mean diameter did so negatively (Figure 7e, Table S6; *p* < 0.01). All these relationships were consistent across global change treatments. Meanwhile, the aboveground biomass differed negligibly amongst treatments (one‐way ANOVA for plots where RWU was measured during our study, *F*(5, 103) = 0.78, *p* = 0.56), indicating that findings were not influenced by treatment differences in canopy water demand. Overall, moderate amounts of *RWU*_*max*_ variability were explained by differences in fine root production and studied traits across the soil profile (Table S6; *R²m =* 0.1–0.4). Notably, the total length of fine roots also promoted *RWU*_*max*_, but this effect resembled that of root mass, which had a stronger impact overall. Any difference in effects of length and mass on *RWU*_*max*_ is reflected through their ratio (SRL).

## 4 Discussion

### 4.1 Root Water Uptake under Global Change

In many regions worldwide, climate warming accelerates the water cycle by elevating VPD (Allan et al. 2020; Wang, Meili, and Fatichi 2023). Therefore, warming should increase grassland RWU where water availability is not limiting, though plants may regulate transpirational water losses by adjusting canopy conductance when VPD exceeds a threshold (Yang et al. 2012; Grossiord et al. 2020; Sadok, Lopez, and Smith 2021). In the studied C_3_ grassland, which is climatically considered non‐water‐limited (Forstner et al. 2023), warming increased fractions of SWC extracted by roots as VPD rose (Figure 4b). However, rooting zones were persistently drier under warming (Figure S4), thus daily RWU was not increased (Figure 2a–h). Daily RWU even decreased at times when warming caused VPD to reach particularly high values (Figures 2d,g and S3) and soil moisture was critically reduced (Figure S4). Although increased stomatal regulation may be expected to underpin these RWU reductions (Grossiord et al. 2020; Yang et al. 2023), canopy conductance (reflected by RWU relative to VPD) remained similar to ambient values (Figures 4d and S5e,f) while roots extracted larger fractions of SWC (Figures 4b and S5b,c). This suggests that the grassland exhibits ‘water‐consumptive’ rather than water‐sparing behaviour at high temperatures, which can support productivity in well‐watered environments (Sadok, Lopez, and Smith 2021; Teuling et al. 2010).

Rising atmospheric CO_2_ concentrations have been linked to plant water savings by permitting downregulation of canopy conductance (De Kauwe, Medlyn, and Tissue 2021; Hatfield and Dold 2019; Liu et al. 2023; Morgan et al. 2011; Roy et al. 2016). This should lead to reduced RWU rates at any VPD. As hypothesized, eCO_2_ reduced the fraction of SWC extracted by roots at high VPD (Figure 4b), reflecting water‐sparing behaviour. Since canopy biomass amongst treatments was similar, this was unlikely to be related to changes in transpiring leaf area, which sometimes affect water dynamics under eCO_2_ (De Kauwe, Medlyn, and Tissue 2021; Walker et al. 2021). However, as grassland was only exposed to high VPD for short periods each day (Figure 5), negligible water sparing (i.e., RWU reductions) accumulated daily (Figures 2a–h and S4). During comparatively warm periods (Bishop, Leakey, and Ainsworth 2014; Morgan et al. 2004; Roy et al. 2016) and experimental warming (Forstner et al. 2023; Habermann et al. 2022; Morgan et al. 2011), eCO_2_ water‐sparing effects have been found to be larger, partly or even fully offsetting water losses due to warming. Accordingly, in our study eCO_2_‐related water‐sparing occurred across broader VPD conditions under warming (Figure 4a), consistent with effects expected under more downregulated canopy conductance (Bachofen et al. 2023; Grossiord et al. 2020). Despite the sometimes wetter rooting zone under these future conditions (compared to warming; Figure S4), which could have reflected a water‐sparing eCO_2_ effect (Morgan et al. 2011; Roy et al. 2016) and might relate to changes in fine roots (see below), eCO_2_ did not clearly alter RWU reductions under warming (Figure 2). Our results—and independent ET observations (Forstner et al. 2021)—thus predominantly support the emerging notion that warming will affect the future water dynamics in C_3_ grassland more strongly than eCO_2_ (Obermeier et al. 2017; Wang, Wang, and Liu 2022; Yuan et al. 2018).

Droughts limit plant access to water and increase sensitivity to VPD (Fu et al. 2022; Koehler et al. 2023). Therefore, the strongest RWU declines under drought are expected with high VPD and warming, and should be alleviated by eCO_2_ (Gampe et al. 2021; Grossiord et al. 2020; Wang, Wang, and Liu 2022). Surprisingly, despite higher VPD under future conditions (Figure S3), drought treatments reduced RWU similarly in 2017 and 2019 (Figure 2a,c), consistent with drought effects of moderate intensity (Yan, Zhong, and Shangguan 2016). However, as soils under future conditions were drier (Figure S4), larger fractions of SWC were extracted by roots to maintain these RWU levels (Figure S5a,b), pointing to an increased threat of water limitation, as previously projected (Denissen et al. 2022). Future conditions aggravated the drought effect on RWU only in 2020 (Figure 2g). In this comparatively hot summer, grassland canopy conductance decreased under persistently high VPD, reflected by earlier and reduced RWU responses to VPD (Figures S5f and 5) which resembled sap flow responses to VPD during an extreme drought in trees (Gimenez et al. 2019). High temperatures—intensified by warming—further elevated VPD during this drought (Figures 1b,c and S3), amplifying RWU reductions under future conditions by 20% (Figure 2g). Such heat‐drought events, often termed hot droughts, intensify water stress by accelerating water losses and advance the onset of heat stress by reducing transpirational cooling, with strong repercussions for plant water dynamics (Bachofen et al. 2023; Barkaoui and Volaire 2023; Sadok, Lopez, and Smith 2021). Altogether, these results highlight that hot droughts, which will occur more frequently in the future (Naumann et al. 2018; Yuan et al. 2019), will severely restrict future grassland water dynamics, with consequences for global water exchange.

To improve water access during drought, grasslands have been suggested to shift water uptake to deeper soil layers, though conflicting studies exist (e.g., Guderle et al. 2018; Deseano Diaz et al. 2023; see introduction for more). Our study found that the fractions of total RWU increased towards deeper soil layers during drought in ambient and future conditions (Figure 3a,c,g), including during the hot drought (Table S2). This provides clear evidence for water‐sourcing shifts in C_3_ grassland, contrasting with some previous findings (Prechsl et al. 2015), and supports the emerging notion that these shifts are maintained as droughts become more severe under warmer future conditions (Weides et al. 2024). The absence of an increase in deep rooting (Figure S7) suggests that these shifts were primarily driven by the top‐down drying of soil.

### 4.2 Fine Rooting and Water Uptake Capacity under Global Change

In our study, fine root responses to global change treatments were less pronounced than previously observed in other grasslands (Arndal et al. 2018; Carrillo et al. 2014; Mueller et al. 2018). It was only warming that increased the ratio of fine root‐to‐shoot production (Figure 6e), as well as fine root diameter and density (Figure 6b,d), and the effect was offset by eCO_2_, as similarly reported for a semiarid grassland (Carrillo et al. 2014). The lack of fine root responses in the eCO_2_ treatment alone might have been due to management‐related fertilization, which likely prevented stimulating effects of nutrient limitation on root growth (Song et al. 2019). By contrast, the interaction of the warming effect with eCO_2_ points towards the possibility that fine root adaptations counteracted intensified soil moisture depletion under warming (Figure 4a and S4), which was alleviated through the water‐sparing effects of eCO_2_ (see above, and Forstner et al. 2023; Habermann et al. 2022; Morgan et al. 2011). Interestingly, the drought events did not alter fine root production and traits (diameters, SRL, SRA), probably because the rapid loss of soil water strongly diminished root growth, and any related adaptive responses (Zhou et al. 2018).

Irrespective of the limited effects of global change treatments on roots, grassland RWU capacity (indicated by *RWU*_*max*_) was generally positively related to the growth and SRL of fine roots, and negatively related to mean root diameter (Figure 7a,b,d), as hypothesized. These relationships highlight the frequently underrated ecohydrological implications of fine root production and traits (Cusack et al. 2024; Freschet et al. 2021), which might play out even more strongly in ecosystems displaying more pronounced root responses to global changes.

### 4.3 Conclusions

Our study shows that in a managed C_3_ grassland, high temperatures, amplified by warming, exacerbate reductions of root water uptake under drought, with negligible water‐sparing effects from eCO_2_. Furthermore, it provides clear evidence that drought, both under current and future (warming, eCO_2_) conditions, shifts root water sourcing towards deeper soil layers. Finally, the overall relationships of grassland water uptake capacity to specific root length and root diameter point towards a so‐far underappreciated role of root traits for grassland water uptake. We conclude that under warmer future conditions, irrespective of shifts in water sourcing, hot droughts will lead to increasingly severe restrictions of grassland water dynamics.

## Conflicts of Interest

The authors declare no conflicts of interest.

## Supporting information

Supporting information.

## Data Availability

All data supporting the findings of this study are available at the ZENODO data repository: https://doi.org/10.5281/zenodo.14142412.
