# Supplementary material for: Individual Versus Combined Effects of Warming, Elevated CO2 and Drought on Grassland Water Uptake and Fine Root Traits
Source: Plant Cell Environ. 2024 Nov 18;48(3):2083–98. doi: 10.1111/pce.15274 (PMC11788968; doi:10.1111/pce.15274)
Supplement: Supplementary file 1 — Supporting information. [file PCE-48-2083-s001.docx]

### Supplementary information for: Individual versus combined effects of warming, elevated CO_2_ and drought on grassland water uptake and fine root traits

**Authors:**

Maud Tissink^1^, Jesse Radolinski^1,2^, David Reinthaler^1^, Sarah Venier^1^, Erich M. Pötsch^3^, Andreas Schaumberger^3^, and Michael Bahn^1^

**Supporting information**: 7349 w. (excl. references)

No of Figures: 7 (all colour)

No of Tables: 6

**S1:** Extended methodology for root water uptake calculation

Root water uptake (RWU) was calculated across the 0–36 cm soil profile using soil water content (θ) measured at 3, 9, 18 and 36 cm depth. To do this, we used the multi-step, multi-layer regression method described by Guderle and Hildebrandt (2015). RWU was derived from the sink term, *S*, in the universal 1-D unsaturated flow, or Richards’ equation:

$$\frac{\partial\theta}{\partial t}= \frac{\partial}{\partial z}\left[ K(h)\left( \frac{\partial p}{\partial z}+1 \right) \right]-S\left( z,t \right)$$

where *z* is the vertical position, *p* is soil matric potential, *K(h)* is the soil unsaturated hydraulic conductivity, and *S(z, t)*, the sink term (water uptake by roots, evaporation, etc.). This method partitions the per-depth change in soil water content over time (∂θ/∂t) into vertical flow (subscript *flow*) and sink (subscript *RWU*) terms from the Richards’ equation:

$\frac{\partial\theta}{\partial t}= \left[ \frac{\partial\theta}{\partial t} \right]_{flow}+\left[ \frac{\partial\theta}{\partial t} \right]_{RWU}$.

During relatively dry weather conditions, strong diurnal fluctuations could be used to distinguish daytime decreases from night-time increases in soil moisture (**Fig. S1**). To do this, we split each day into daytime and night-time using the standard Austrian sunrise and sunset times throughout the year. During periods with fluctuations, a zero flux plain (Khalil et al., 2003) develops in the soil profile, above which soil water rises due to strongly negative potentials at the surface, whereas downward flow dominates below this point.

We attributed decreases in θ during the daytime to vertical flow and evapotranspiration (ET), which included direct abstraction by the plant for transpiration (*∂θ/∂t_flow_ + ∂θ/∂t_RWU_*) and subsequent soil water flow. Potential gradients in the soil profile persist overnight driving soil water flow upward (Chanzy et al., 2012; Verhoef et al., 2006); however, soil water extraction is negligible as solar energy decreases, plants close their stomata, and transpiration falls to zero (Loheide II, 2008). Under the assumption that ET is negligible at night and that night-time soil water flow occurs at a rate indistinguishable from daytime values we considered the change in soil moisture to be solely derived from vertical flow at night (*∂θ/∂t_flow_*).

Previous work (Chai et al., 2023; Jackisch et al., 2020; Lai et al., 2023; Li et al., 2002; Renner et al., 2016) has shown that fitting linear models to the day- and night-time components of these diurnal cycles can sufficiently describe RWU and flow fluxes. We applied robust linear models (C. Yu & Yao, 2017), representing trends during daytime with *m_day_* and night-time with *m_night_*. By using M-estimation, robust approaches assign higher weight to data with smaller residuals and lower weight to data with larger residuals, making models less sensitive to outliers compared with the frequently used least-squares approach. This minimised the influence of inflections at sunrise and sundown. With sufficient depth resolution of rootzone soil moisture dynamics, uptake estimates per depth *z* can be integrated into a single estimate of uptake *m_RWU_* for the studied control volume:

$$m_{RWU}=n\left( m_{day}+m_{night} \right)\partial z$$

where *n* is the number of daylight hours for daily estimates [mm day^-1^] or *n* = 1 for hourly estimates [mm hour^-1^]. To capture hourly variation, the main part of this paper describes in more detail the replacement of the linear *m_day_* with a robust third-degree polynomial model (see **Fig. S1**). This estimates *m_RWU_* for each daylight hour and improves resolution during periods where data is sufficiently available.

The proposed method has been shown to be an accurate estimate of RWU in vegetated soil profiles because 1) strong diurnal soil moisture fluctuations in soil moisture have an overwhelming influence from plant water extraction (Jackisch et al., 2020; Schelde et al., 2011; White, 1932) and 2) if isolated correctly, periods without rapid water flow accentuate these signatures. Thus, this method produces comparable and often more reliable results than more complex hydraulic methods which solve traditional flow equations or derived uptake from inverse solutions (Guderle & Hildebrandt, 2015). The contribution of transpiration to ET in grasslands often approaches 100% during the growing season (Dubbert et al., 2013; Good et al., 2014; Quade et al., 2019). On this basis, we assumed that most detected activity is the result of plant transpiration, and our RWU metric could describe plant driven acquisition strategies between climate change scenarios.

**S2:** Extended methodology for statistical analyses

To analyse the effects of global change treatments on response variables, we employed linear mixed-effects models using the ‘lmer’ function in R (*lme4* package; Bates *et al.*, 2015; Bolker, 2024). The model was generally formulated as ‘lmer (response variable ∼ treatment × other + (1|time) + (1|space), data = data)’. The response variables in our models were daily RWU, fractions of RWU across the main rooting horizon, RWU_SWC_ and RWU_VPD_, fine root production and traits, the ratio of fine root- to shoot production, and *RWU_max_*. Fixed effects were global change treatments and any other parameter(s) required to constrain any treatment effects (as indicated by ‘other’), as well as their interaction. Examples of these ‘other’ fixed effects include 1) the year and period of the growing season, which were often required to assess drought effects during versus outside rainfall exclusion, and 2) fine root properties (the mass density of roots produced and their trait values), for which we wanted to know how they related to *RWU_max_*. Random intercept effects—denoted by ‘(1|…)’ syntax—accounted for variability over time (period of the growing season nested within years, or daily/hourly variation) and space (plots, grouped by location across the experiment to assess spatial balance), minimizing pseudoreplication (Metze et al., 2023). Across models, the variance of random spatial effects was small compared to residual variance (the former composing largely < 2% of the latter; **Table S1-6**), indicating minimal spatial imbalance across the experiment and that plots can be considered ‘true’ treatment replicates (Meeran et al., 2021). Random time effects ensured that any treatment effects were derived from consistent responses, compensating for the sometimes-limited treatment replication.

We used restricted maximum likelihood estimation to estimate the fixed and random effects coefficients in our models. Statistically insignificant interaction terms were removed where this lowered the Akaike information criterion, improving the balance between model complexity and fit. This only affected models where the response variable was *RWU_max_* and predictor variables included fine root traits; the interaction terms in all other models improved this balance and, thus, retained, resulting in the final model specifications (**S2**). Model fits were assessed using the marginal *R^2^* (*R²m*, the proportion of variance explained by fixed effects) and conditional *R^2^* (*R²c*, the proportion of variance explained by fixed and random effects) (Nakagawa & Schielzeth, 2013), derived using the function ‘r.squaredGLMM’ (*MuMIn* package; Bartoń, 2024). This resulted in the final model specifications, discussed below.

To determine how daily RWU in the main rooting horizon varied across treatments and periods of the growing seasons, we used ‘lmer(log (daily RWU) ∼ treatment × (year/period) + (1∣ location/plot) + (1∣date), data = data)’, using the log transformation to normalize RWU distributions. The year and period of the growing season were included, most importantly, to distinguish between drought effects during and outside rainfall exclusions. Fractions of total RWU across the soil profile, aggregated by depth to ensure independence of fractions of total RWU across the soil profile, were assessed using the same model structure without transformation.

To evaluate how SWC and VPD interactively affected hourly RWU estimates, data were aggregated into SWC and VPD deciles which were analysed independently (Bachofen et al., 2023; T. Yu et al., 2022). RWU responses to SWC and VPD were then compared between treatments from two perspectives: across VPD deciles, we compared RWU_SWC_ (RWU/SWC), indicating the fraction of SWC taken up by roots. Conversely, across SWC deciles, we compared RWU_VPD_ (RWU/VPD), indicating RWU relative to VPD. Effects of treatments on RWU_SWC_ aggregated by VPD decile, and on RWU_VPD_ aggregated by SWC decile, were determined using ‘lmer(RWU_SWC_ ∼ treatment × VPD + (1|date_and_hour), data = data)’ and ‘lmer(RWU_VPD_ ∼ treatment × SWC + (1|date_and_hour) , data = data)’, respectively.

Fine root production, biomass allocation and traits in the main rooting horizon were determined across treatments using ‘lmer(fine root property ∼ treatment + (1| location/plot) + (1|year/period), data = data)’. To analyse how treatment effects on fine root production and traits varied across soil layers, this model was adapted by adding a fixed, interacting ‘soil layer’ term and a random ‘(1|core_id)’ term, referencing the identities of ingrowth cores, again limiting pseudoreplication. Lastly, to determine how these fine root production and traits related to *RWU_max_*, we used ‘lmer(*RWU_max_* ∼ treatment × fine root property × soil layer + (1|location/plot) + (1|core_id), data = data)’. The term ‘soil layer’ was excluded from the fine root production model due to collinearity. Furthermore, interactions between fixed effects in models based on traits (SRL, SRA and mean diameter) were not statistically significant and were removed, which lowered the Akaike information criterion. Visual inspection of the data confirmed that this was due to a combination of 1) highly similar trait values across the soil profile (**Fig. S7**), 2) similar relationships between these traits and *RWU_max_* in each treatment and soil layer, and 3) no change in the effects of global change treatments with depth.

The contribution of global change treatments to the overall variability of each response variable was evaluated using *F*-values obtained using a type III ANOVA table with Satterthwaite’s method (‘Anova’ function, *car* package; Fox, 2023). To compare data distributions amongst treatments, marginal means were extracted using the ‘emmeans’ function (*emmeans* package; Lenth, 2024) and compared with the ‘cld’ function (*multcomp* package; Hothorn, 2023) using α = 0.05 and false discovery rate correction for multiple testing (e.g., six treatments). To meet the assumptions of normality in linear mixed effects modelling, we confirmed the normality of model residuals using Q-Q plots. Residuals plotted against fitted values showed no clear patterns, indicating reliable coefficient estimation (homoscedasticity, no outliers). All statistical analyses were conducted using R software version 4.3.2 (R Core Team, 2023).


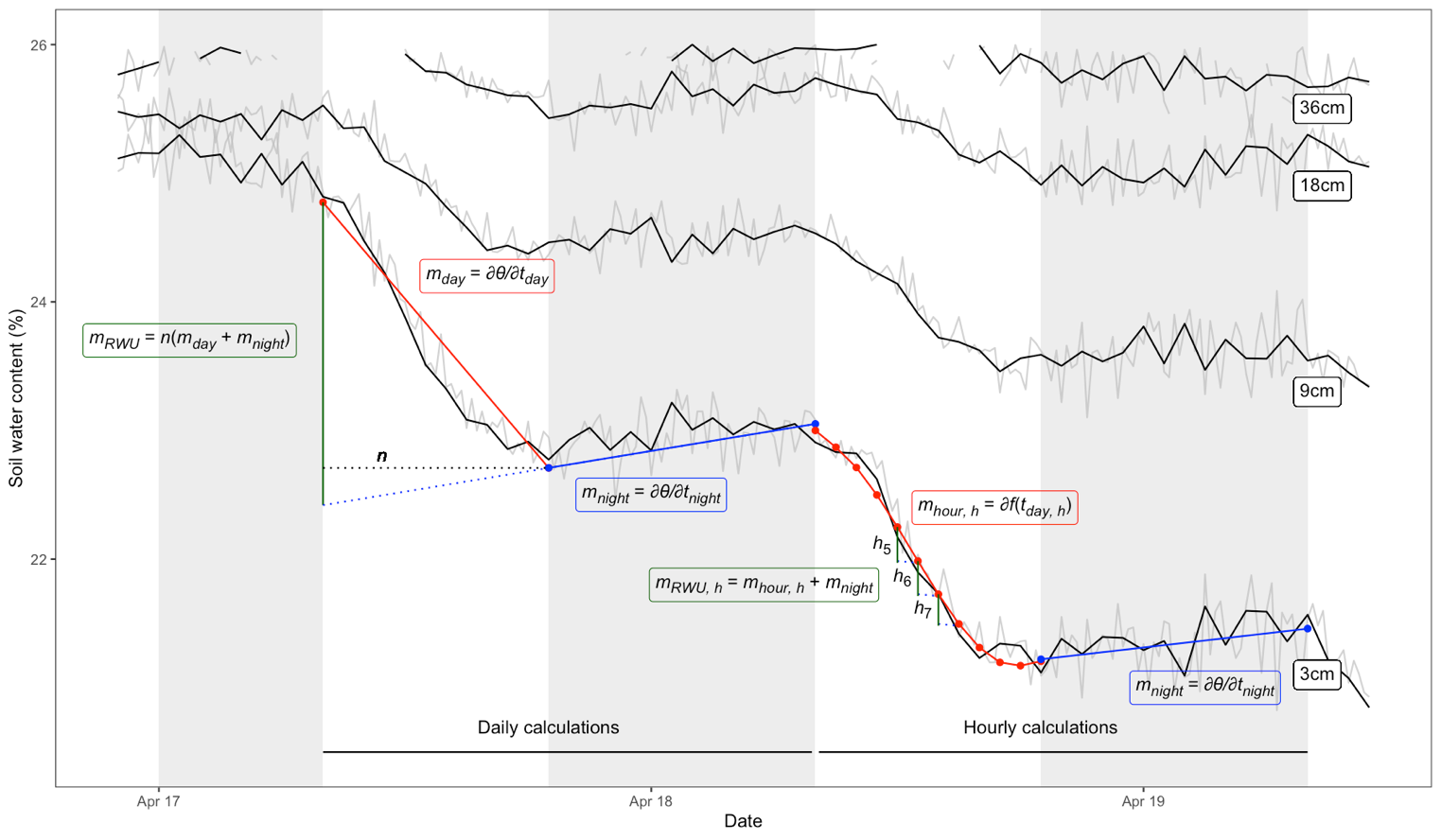


**Figure S1:** Visualisation of root water uptake (RWU) calculations at daily and hourly timescales using soil water content at four depths (3, 9, 18 and 36 cm). Data shown are from a grassland plot under ambient conditions in 2019. Soil water content values at 9, 18 and 36 cm depths were shifted for a condensed view and are shown before (*grey*; raw 15-minute resolution) and after smoothing (*black; hourly medians*). Robust linear models represent soil water content trends during day (*red*) and night (*blue, solid line*), and grassland RWU (*green*) is estimated by adjusting daytime change over n elapsed daytime hours for rates of night-time change (*blue, dotted line*). In hourly calculations, *h* is a daytime hour between 1 and *n*.

**
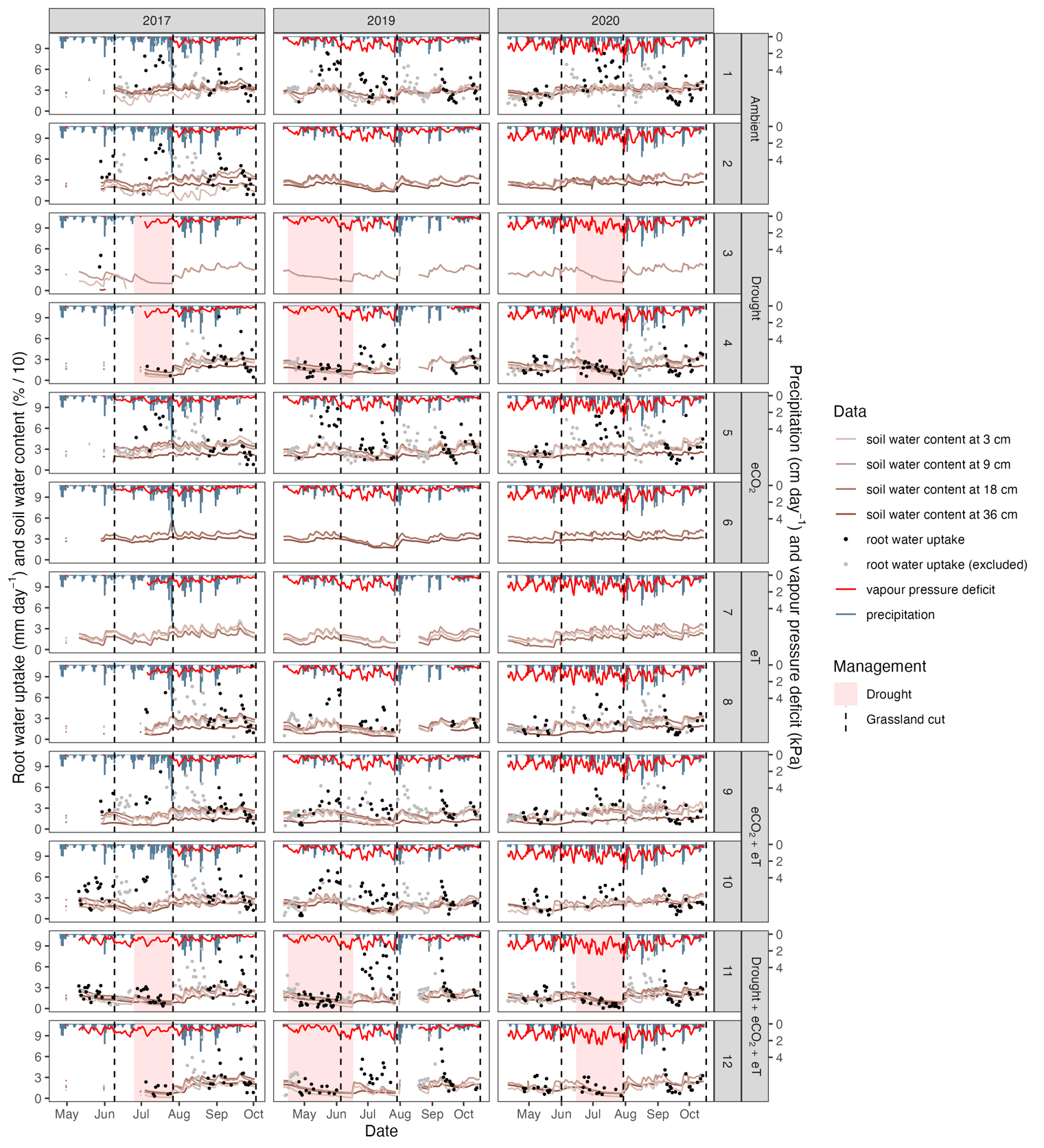
**

**Figure S2:** Root water uptake (RWU), calculated from soil water content (SWC) at four depths (3, 9, 18 and 36 cm), vapour pressure deficit (VPD) and daily precipitation in 12 plots of grassland exposed to individual and combined treatments of warming (eT; +3 °C), elevated CO_2_ (eCO_2_; +300 ppm) and drought over three growing seasons. RWU estimates following snow cover and grassland cuts were excluded from analyses (see methods).


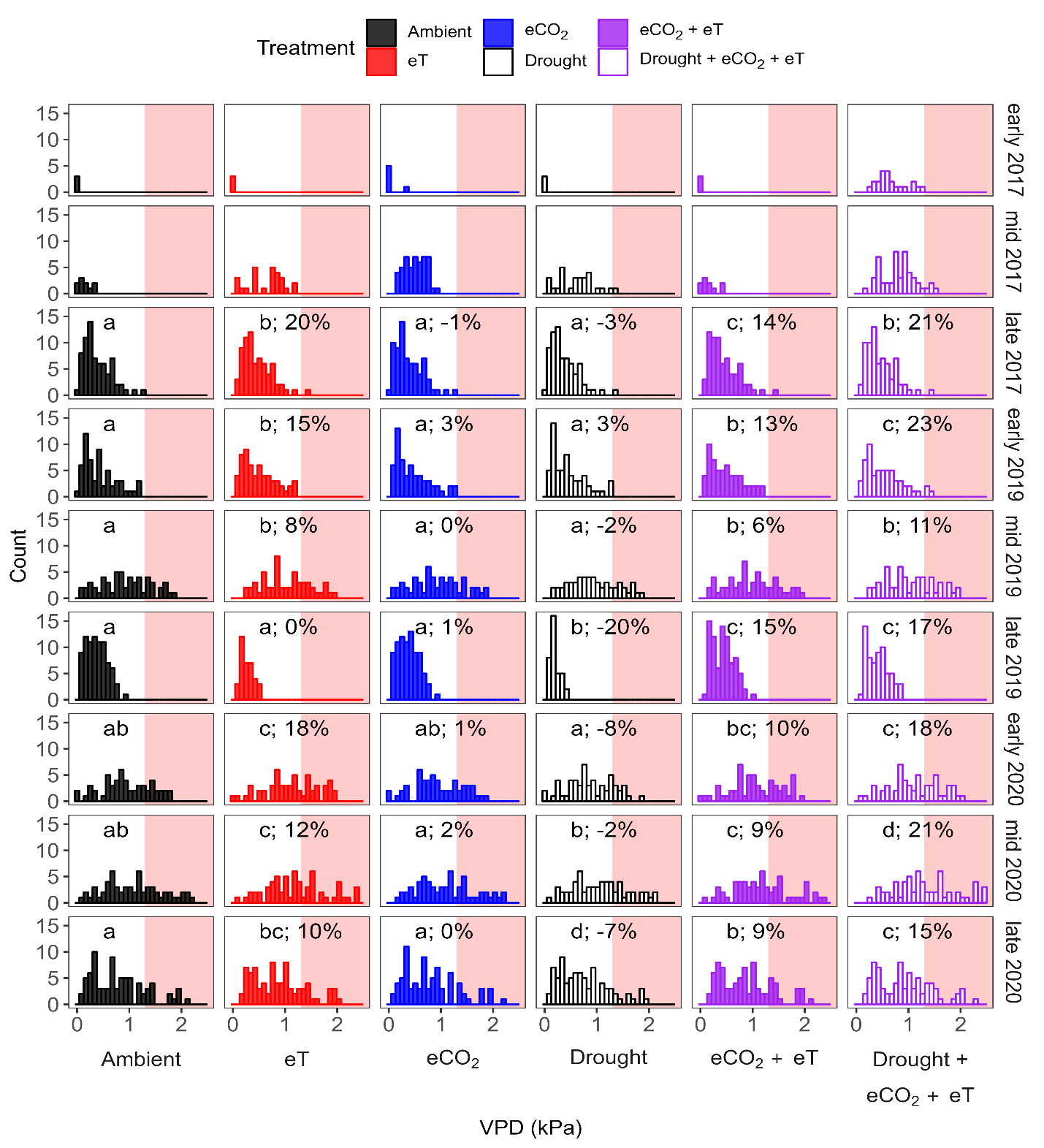


**Figure S3:** Frequency distributions of the mean daily vapour pressure deficit (VPD) in a grassland exposed to individual and combined treatments of warming (eT; +3 °C), elevated CO_2_ (eCO_2_; +300 ppm) and drought across stages of the three growing seasons. Red shading indicates the high VPD range (> 1.3 kPa). Treatment distributions not sharing any letter are different at the 5% level of significance and percentages show treatment effects. Note that treatments were not compared during the early- to mid-season of 2017 as data were not sufficiently available (**Fig. 1**, **Fig. S2**).

**
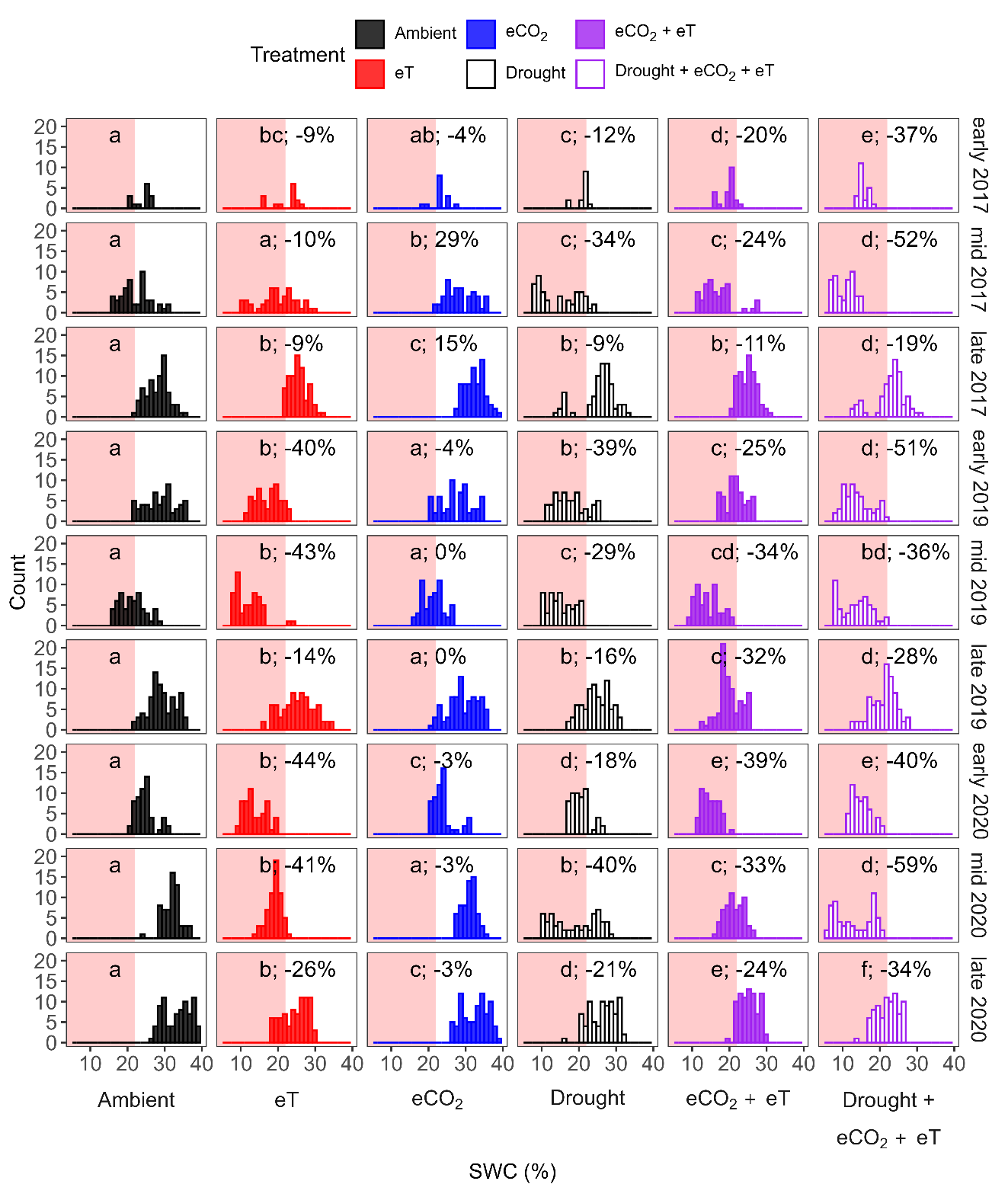
**

**Figure S4:** Frequency distributions of the mean daily soil water content (SWC) in a grassland exposed to individual and combined treatments of warming (eT; +3 °C), elevated CO_2_ (eCO_2_; +300 ppm) and drought across stages of the three growing seasons. Red shading indicates the low SWC range (< 22%). Treatment distributions not sharing any letter are different at the 5% level of significance and percentages show treatment effects.


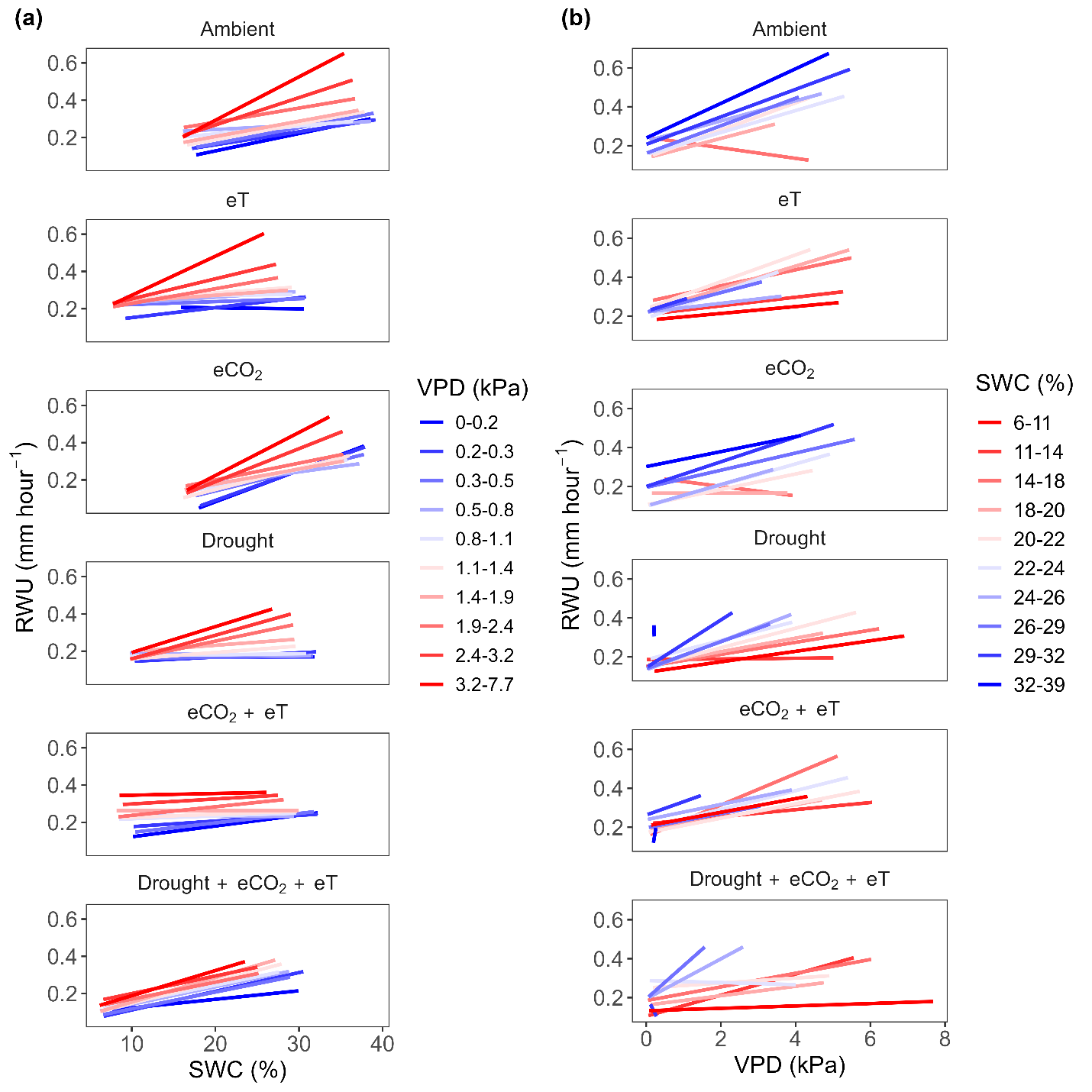


**Figure S5:** Linear models showing effects of soil water content (SWC) and vapour pressure deficit (VPD) on hourly root water uptake (RWU) in grassland exposed to individual and combined treatments of warming (eT; +3 °C), elevated CO_2_ (eCO_2_; +300 ppm), and drought. The panels show (**a**) SWC effects on RWU for each treatment across VPD deciles and (**b**) VPD effects on RWU for each treatment across SWC deciles.


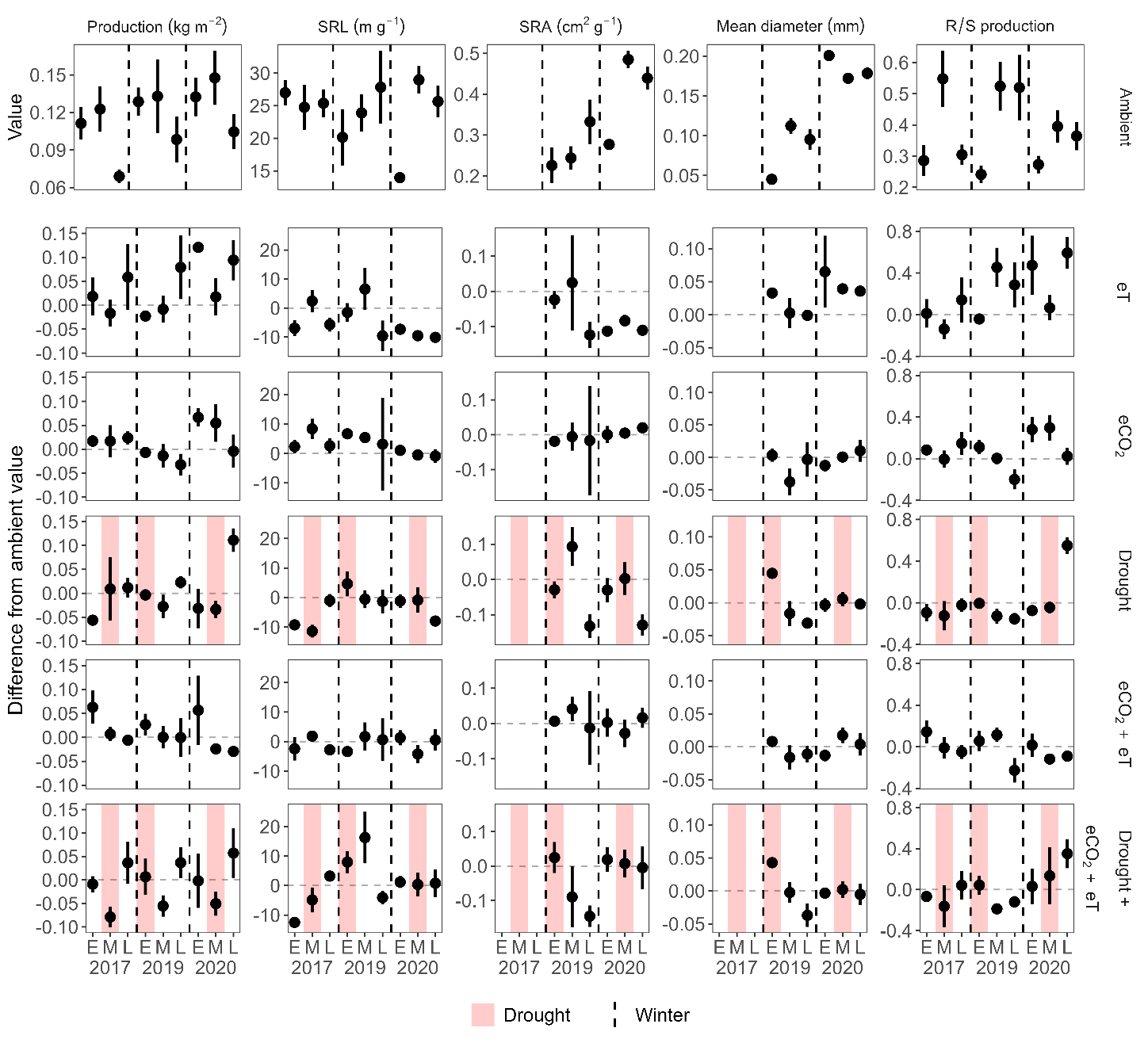


**Figure S6:** Mean fine root production, traits (SRL = specific root length; SRA = specific root area) and the ratio of fine root- to shoot production (R/S production) in a grassland exposed to individual and combined treatments of warming (eT; +3 °C), elevated CO_2_ (eCO_2_; +300 ppm) and drought over three growing seasons. Error bars show standard errors. Roots were extracted three times per growing season (E = early; M = mid; L = late) in 2017, 2019 and 2020.

**
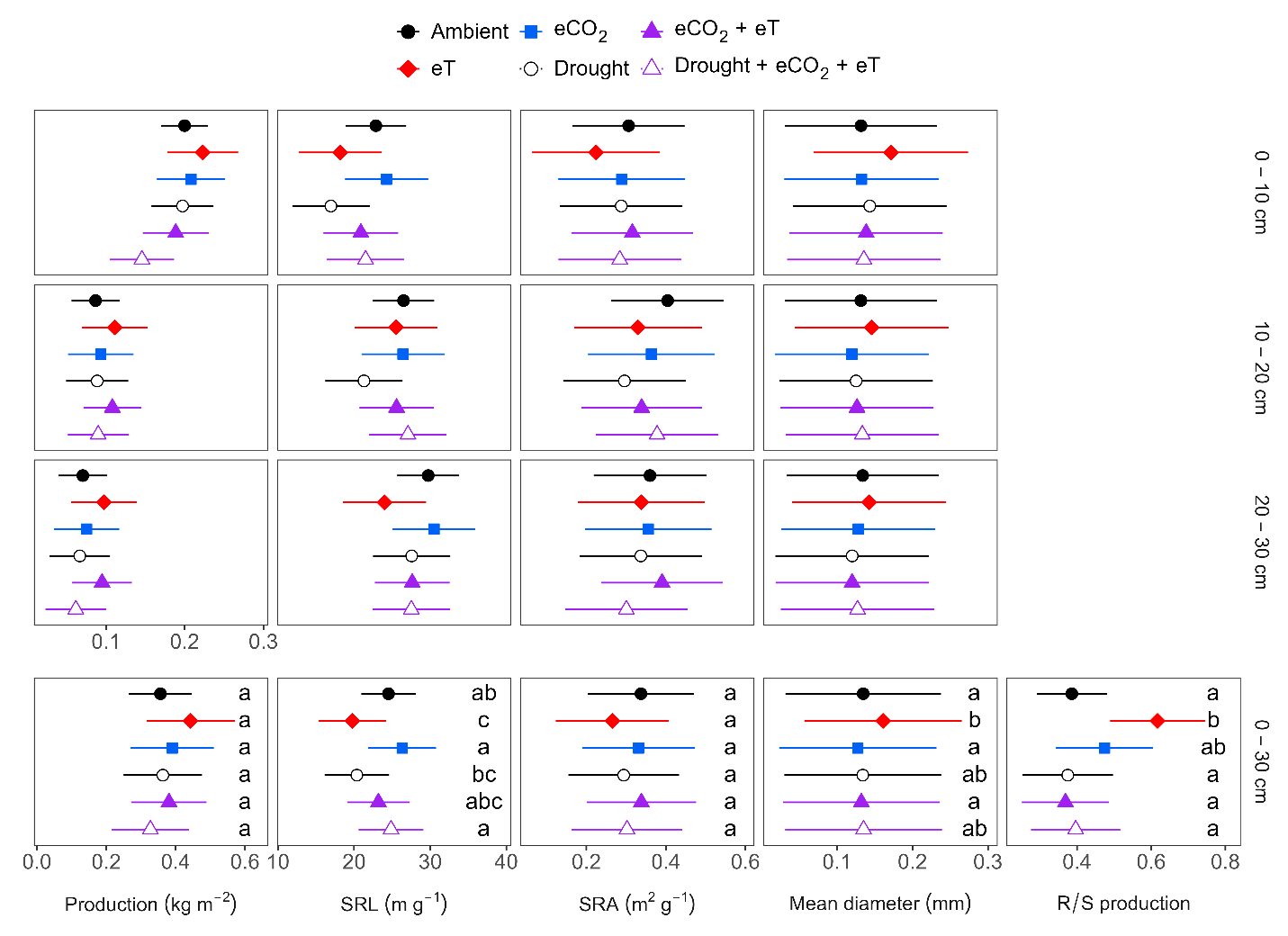
**

**Figure S7:** Mean annual production of fine roots and their traits (where SRL = specific root length; SRA = specific root area) in three soil layers, and the ratio of fine root- to shoot production (R/S production), in a grassland exposed to individual and combined treatments of warming (eT; +3 °C), elevated CO_2_ (eCO_2_; +300 ppm), and drought. For change over time, see **Fig. S6**. Error bars denote 95% confidence intervals around the marginal means. Values not sharing any letter within facets differ at the 5% significance level (no letters mean no differences). Roots were extracted three times per growing season in 2017, 2019 and 2020.

**Table S1:** Outputs for the linear mixed-effect model specified as ‘Log (RWU) ∼ Treatment × (Year/Period) + (1∣Location/Plot) + (1∣Date)’. For details, refer to **S2**. In the ‘fixed effects’ section, ‘output’ shows R outputs generated using the ‘anova’ function. In the ‘random effects’ section, ‘estimates’ refer to variances of residuals and specific random effects, as well as sample numbers. For the model, total observations as well as marginal and conditional *R*-squared values (*R^2^m*, *R^2^c*) are shown. Treatment pair differences at the 5% level of significance are shown. *P*-values were adjusted for multiple testing using false discovery rate correction.

|  | Log-transformed RWU |
| --- | --- |
| Fixed Effects | *Output* |
| treatment  year  year:period  treatment:year  treatment:year:period | Sum Sq Mean Sq NumDF DenDF F value Pr(>F)  19.659 3.9317 5 45.97 47.9781 < 2.2e-16 ***  0.975 0.4875 2 198.72 5.9490 0.003096 **  0.915 0.1525 6 191.58 1.8613 0.089400 .  13.607 1.3607 10 731.43 16.6043 < 2.2e-16 ***  59.608 2.1289 28 785.81 25.9779 < 2.2e-16 *** |
| Random Effects | *Estimates* |
| σ^2^ | 0.08 |
| τ_00_ _date_ | 0.29 |
| τ_00_ _plot:location_ | 0.00 |
| τ_00_ _location_ | 0.00 |
| ICC | 0.78 |
| N _date_ | 205 |
| N _plot_ | 10 |
| N _location_ | 4 |
| Model | *Values* |
| Observations | 1028 |
| *R^2^m* / *R^2^c* | 0.343 / 0.857 |
| Pair differences where *p* < 0.05 | |
| year = 2017, period = early:  contrast estimate SE df t.ratio p.value  Ambient - (Drought + eCO2 + eT) 1.1158002 0.2598126 21.25 4.295 0.0009  Drought - (Drought + eCO2 + eT) 0.6505639 0.2574995 118.41 2.526 0.0257  (eCO2 + eT) - (Drought + eCO2 + eT) 0.8253732 0.1737515 14.02 4.750 0.0009  year = 2019, period = early:  contrast estimate SE df t.ratio p.value  Ambient - Drought 1.2343205 0.1939477 14.12 6.364 0.0001  Ambient - (eCO2 + eT) 0.4214321 0.1266670 45.53 3.327 0.0037  Ambient - (Drought + eCO2 + eT) 1.2878177 0.1561942 26.78 8.245 <.0001  eT - Drought 1.0783168 0.1963716 7.29 5.491 0.0020  eT - (Drought + eCO2 + eT) 1.1318139 0.1508661 14.58 7.502 <.0001  eCO2 - Drought 1.3063349 0.2138582 7.82 6.108 0.0009  eCO2 - (eCO2 + eT) 0.4934465 0.1491331 13.42 3.309 0.0102  eCO2 - (Drought + eCO2 + eT) 1.3598321 0.1800756 10.69 7.551 0.0001  Drought - (eCO2 + eT) -0.8128884 0.1820721 2.45 -4.465 0.0474  (eCO2 + eT) - (Drought + eCO2 + eT) 0.8663856 0.1261117 2.64 6.870 0.0157  year = 2017, period = mid:  contrast estimate SE df t.ratio p.value  Ambient - Drought 1.4110980 0.2732728 38.92 5.164 <.0001  Ambient - (Drought + eCO2 + eT) 1.3670185 0.1827091 11.71 7.482 <.0001  eT - Drought 1.0174896 0.2690016 42.02 3.782 0.0009  eT - (Drought + eCO2 + eT) 0.9734101 0.2074079 61.47 4.693 <.0001  eCO2 - Drought 1.4713804 0.2846926 50.81 5.168 <.0001  eCO2 - (Drought + eCO2 + eT) 1.4273009 0.2041651 22.58 6.991 <.0001  Drought - (eCO2 + eT) -1.2702988 0.2571172 29.98 -4.941 0.0001  (eCO2 + eT) - (Drought + eCO2 + eT) 1.2262193 0.1631222 14.56 7.517 <.0001  year = 2019, period = mid:  contrast estimate SE df t.ratio p.value  Ambient - eT 0.4975514 0.1611622 19.03 3.087 0.0454  eT - (Drought + eCO2 + eT) -0.7558789 0.1289120 6.71 -5.864 0.0109  year = 2020, period = mid:  contrast estimate SE df t.ratio p.value  Ambient - eT 0.4521529 0.1581267 16.98 2.859 0.0181  Ambient - Drought 1.0767272 0.1881910 9.75 5.721 0.0006  Ambient - (eCO2 + eT) 0.4755314 0.1241641 40.95 3.830 0.0011  Ambient - (Drought + eCO2 + eT) 1.6211495 0.1525170 23.72 10.629 <.0001  eT - eCO2 -0.4621027 0.1812643 8.63 -2.549 0.0435  eT - Drought 0.6245743 0.1773865 3.22 3.521 0.0435  eT - (Drought + eCO2 + eT) 1.1689966 0.1282379 6.60 9.116 0.0003  eCO2 - Drought 1.0866770 0.2100358 5.75 5.174 0.0050  eCO2 - (eCO2 + eT) 0.4854812 0.1482572 12.29 3.275 0.0121  eCO2 - (Drought + eCO2 + eT) 1.6310993 0.1780521 10.07 9.161 <.0001  Drought - (Drought + eCO2 + eT) 0.5444223 0.1090470 2.84 4.993 0.0264  (eCO2 + eT) - (Drought + eCO2 + eT) 1.1456181 0.1355996 6.37 8.449 0.0004  Degrees-of-freedom method: kenward-roger  Results are given on the log (not the response) scale.  P value adjustment: fdr method for varying numbers of tests | |
|  | |

**Table S2:** Outputs for the linear mixed-effect model specified as ‘lmer(fraction of RWU ∼ treatment × (year/period) + (1∣location/plot) + (1∣date), data = data)’, presented in percentage format. For details, refer to **S2**. In the ‘fixed effects’ section, ‘output’ shows R outputs generated using the ‘anova’ function. In the ‘random effects’ section, ‘estimates’ refer to variances of residuals and specific random effects, as well as sample numbers. For the model, total observations as well as marginal and conditional *R*-squared values (*R^2^m*, *R^2^c*) are shown. Treatment pair differences at the 5% level of significance are shown. *P*-values were adjusted for multiple testing using false discovery rate correction.

|  | % at 3 cm | % at 9 cm | % at 18 cm | % at 36 cm |
| --- | --- | --- | --- | --- |
| Fixed Effects | *Output* | *Output* | *Output* | *Output* |
| Treatment  year  year:period  treatment:year  treatment:year:period | F Pr(>F)  10.65 0.02  7.10 0.00  5.74 0.00  2.52 0.01  4.99 0.00 | F Pr(>F)  8.93 0.00  0.21 0.81  0.60 0.73  7.63 0.00  5.34 0.00 | F Pr(>F)  11.56 0.00  6.88 0.00  6.30 0.00  1.80 0.06  3.19 0.00 | F Pr(>F)  21.88 0.00  0.59 0.55  2.97 0.01  3.33 0.00  7.57 0.00 |
| Random Effects | *Estimates* | *Estimates* | *Estimates* | *Estimates* |
| σ^2^ | 2.91 | 0.89 | 0.49 | 0.50 |
| τ_00_ | 1.76 _date_ | 0.39 _date_ | 0.23 _date_ | 0.47 _date_ |
|  | 0.04 _plot:location_ | 0.00 _plot:location_ | 0.00 _plot:location_ | 0.00 _plot:location_ |
|  | 0.00 _location_ | 0.12 _location_ | 0.00 _location_ | 0.00 _location_ |
| N | 215 _date_ | 215 _date_ | 215 _date_ | 215 _date_ |
|  | 10 _plot_ | 10 _plot_ | 10 _plot_ | 10 _plot_ |
|  | 4 _location_ | 4 _location_ | 4 _location_ | 4 _location_ |
| Model | *Values* | *Values* | *Values* | *Values* |
| Observations | 1047 | 1047 | 1047 | 1047 |
| *R^2^m* / *R^2^c* | 0.332 / 0.53 | 0.273 / 0.49 | 0.261 / 0.45 | 0.356 / 0.60 |
| Pair differences at 3 cm where *p* < 0.05 | | | | |
| year = 2017, period = early:  contrast estimate SE df t.ratio p.value  Ambient - (eCO2 + eT) -2.588581 1.0472491 124.30 -2.472 0.0444  (eCO2 + eT) - (Drought + eCO2 + eT) 3.275772 0.7281370 20.10 4.499 0.0013  year = 2019, period = early:  contrast estimate SE df t.ratio p.value  eT - (Drought + eCO2 + eT) 2.779102 0.9394626 42.88 2.958 0.0376  (eCO2 + eT) - (Drought + eCO2 + eT) 1.986417 0.5802281 15.00 3.424 0.0376  year = 2020, period = early:  contrast estimate SE df t.ratio p.value  Ambient - (Drought + eCO2 + eT) 2.433403 0.7102778 62.25 3.426 0.0164  eCO2 - (Drought + eCO2 + eT) 2.266487 0.7145045 41.24 3.172 0.0214  year = 2017, period = mid:  contrast estimate SE df t.ratio p.value  Ambient - eCO2 2.434538 0.8502971 38.03 2.863 0.0170  Ambient - Drought 3.768951 1.2279546 310.27 3.069 0.0070  Ambient - (Drought + eCO2 + eT) 3.249529 0.7837315 92.40 4.146 0.0006  eT - (Drought + eCO2 + eT) 2.992900 1.2359686 147.58 2.422 0.0357  eCO2 - (eCO2 + eT) -3.161056 0.9675648 129.57 -3.267 0.0052  Drought - (eCO2 + eT) -4.495469 1.3123477 311.34 -3.426 0.0035  (eCO2 + eT) - (Drought + eCO2 + eT) 3.976047 0.7998480 67.43 4.971 0.0001  year = 2019, period = mid:  contrast estimate SE df t.ratio p.value  Ambient - (eCO2 + eT) 2.561223 0.6248296 32.79 4.099 0.0038  year = 2020, period = mid:  contrast estimate SE df t.ratio p.value  Ambient - Drought 2.983704 0.7698338 30.29 3.876 0.0027  Ambient - (Drought + eCO2 + eT) 2.264166 0.6533873 40.62 3.465 0.0047  eT - Drought 3.321357 1.1732390 39.33 2.831 0.0165  eT - (Drought + eCO2 + eT) 2.601819 0.8129825 20.61 3.200 0.0131  eCO2 - Drought 2.349681 0.8148027 26.67 2.884 0.0165  eCO2 - (Drought + eCO2 + eT) 1.630142 0.6899499 33.48 2.363 0.0452  Drought - (eCO2 + eT) -3.623246 0.8813112 37.86 -4.111 0.0015  (eCO2 + eT) - (Drought + eCO2 + eT) 2.903707 0.6483781 31.46 4.478 0.0014 | | | | |
| Pair differences at 18 cm where *p* < 0.05 | | | | |
| year = 2019, period = early:  contrast estimate SE df t.ratio p.value  eCO2 - Drought -0.9227492 0.3335480 61.39 -2.766 0.0422  eCO2 - (Drought + eCO2 + eT) -0.7670262 0.2823244 64.76 -2.717 0.0422  Drought - (eCO2 + eT) 0.8659204 0.3256741 29.46 2.659 0.0471  (eCO2 + eT) - (Drought + eCO2 + eT) -0.7101974 0.2304272 16.33 -3.082 0.0422  year = 2017, period = mid:  contrast estimate SE df t.ratio p.value  Ambient - Drought -1.9166951 0.5025406 340.03 -3.814 0.0008  Ambient - (Drought + eCO2 + eT) -0.7917617 0.3110141 104.17 -2.546 0.0265  eT - Drought -2.1075085 0.6171667 280.89 -3.415 0.0027  eCO2 - Drought -1.2622956 0.5239263 348.68 -2.409 0.0309  eCO2 - (eCO2 + eT) 1.2390497 0.3719854 227.27 3.331 0.0030  Drought - (eCO2 + eT) 2.5013452 0.5175163 241.14 4.833 <.0001  Drought - (Drought + eCO2 + eT) 1.1249334 0.4312813 192.53 2.608 0.0245  (eCO2 + eT) - (Drought + eCO2 + eT) -1.3764118 0.3222410 71.98 -4.271 0.0004  year = 2020, period = mid:  contrast estimate SE df t.ratio p.value  Ambient - Drought -0.9414501 0.3001233 45.95 -3.137 0.0112  eT - eCO2 -0.9198898 0.3418539 62.87 -2.691 0.0228  eT - Drought -1.6153853 0.3805700 25.94 -4.245 0.0019  eT - (Drought + eCO2 + eT) -1.0018591 0.2894348 27.81 -3.461 0.0088  eCO2 - (eCO2 + eT) 0.6545713 0.2832553 72.01 2.311 0.0451  Drought - (eCO2 + eT) 1.3500668 0.3316324 34.94 4.071 0.0019  Drought - (Drought + eCO2 + eT) 0.6135263 0.2423491 13.89 2.532 0.0451  (eCO2 + eT) - (Drought + eCO2 + eT) -0.7365405 0.2572640 41.71 -2.863 0.0196 | | | | |
| Pair differences at 36 cm where *p* < 0.05 | | | | |
| year = 2017, period = early:  contrast estimate SE df t.ratio p.value  (eCO2 + eT) - (Drought + eCO2 + eT) -0.8933571 0.2979977 18.20 -2.998 0.0459  year = 2019, period = early:  contrast estimate SE df t.ratio p.value  Ambient - (eCO2 + eT) 0.7045005 0.2633247 118.39 2.675 0.0213  Ambient - (Drought + eCO2 + eT) -0.6365903 0.2609500 101.59 -2.440 0.0308  eT - Drought -1.0938833 0.4371270 140.94 -2.502 0.0289  eT - (Drought + eCO2 + eT) -1.1147700 0.3529287 91.40 -3.159 0.0064  eCO2 - Drought -1.2114999 0.3173346 92.90 -3.818 0.0009  eCO2 - (Drought + eCO2 + eT) -1.2323866 0.2697727 94.59 -4.568 0.0001  Drought - (eCO2 + eT) 1.3202041 0.3087605 66.20 4.276 0.0003  (eCO2 + eT) - (Drought + eCO2 + eT) -1.3410907 0.2204828 28.68 -6.083 <.0001  year = 2020, period = early:  contrast estimate SE df t.ratio p.value  Ambient - eT -0.9594693 0.3484488 137.44 -2.754 0.0251  Ambient - (Drought + eCO2 + eT) -0.7649322 0.2720149 129.59 -2.812 0.0251  eT - eCO2 1.0152905 0.3493462 116.15 2.906 0.0251  eCO2 - (Drought + eCO2 + eT) -0.8207534 0.2699974 103.23 -3.040 0.0251  year = 2017, period = mid:  contrast estimate SE df t.ratio p.value  Ambient - (Drought + eCO2 + eT) -1.2080857 0.3086516 191.72 -3.914 0.0009  eT - (Drought + eCO2 + eT) -1.4577846 0.4890381 300.30 -2.981 0.0117  eCO2 - (Drought + eCO2 + eT) -1.5372249 0.3439183 218.09 -4.470 0.0002  (eCO2 + eT) - (Drought + eCO2 + eT) -1.0727463 0.3238944 121.48 -3.312 0.0061  year = 2019, period = mid:  contrast estimate SE df t.ratio p.value  Ambient - (eCO2 + eT) -1.2737686 0.2353392 68.40 -5.412 <.0001  eCO2 - (eCO2 + eT) -0.9236709 0.2565799 71.00 -3.600 0.0022  Drought - (eCO2 + eT) -1.2595983 0.3456483 89.20 -3.644 0.0022  (eCO2 + eT) - (Drought + eCO2 + eT) 1.2005763 0.2289180 27.73 5.245 0.0001  year = 2020, period = mid:  contrast estimate SE df t.ratio p.value  Ambient - Drought -1.1302145 0.2867960 65.48 -3.941 0.0004  Ambient - (Drought + eCO2 + eT) -1.5667779 0.2473734 85.01 -6.334 <.0001  eT - Drought -1.0446636 0.3825352 59.20 -2.731 0.0156  eT - (Drought + eCO2 + eT) -1.4812271 0.2935148 44.16 -5.047 <.0001  eCO2 - Drought -1.3655070 0.2978580 65.97 -4.584 0.0001  eCO2 - (Drought + eCO2 + eT) -1.8020705 0.2614067 85.60 -6.894 <.0001  Drought - (eCO2 + eT) 1.2822828 0.3163577 76.74 4.053 0.0003  (eCO2 + eT) - (Drought + eCO2 + eT) -1.7188463 0.2509501 74.51 -6.849 <.0001 | | | | |
|  | | | | |

**Table S3:** Outputs for the linear mixed-effect model specified as ‘lmer(RWU_SWC_ ~ treatment × SWC + (1|date_and_hour), data = data)’. For details, refer to **S2**. In the ‘fixed effects’ section, ‘output’ shows R outputs generated using the ‘anova’ function. In the ‘random effects’ section, ‘estimates’ refer to variances of residuals and specific random effects, as well as sample numbers. For the model, total observations as well as marginal and conditional *R*-squared values (*R^2^m*, *R^2^c*) are shown. Treatment pair differences at the 5% level of significance are shown. *P*-values were adjusted for multiple testing using false discovery rate correction.

|  | VPD (kPa) | | | | | | | | | |
| --- | --- | --- | --- | --- | --- | --- | --- | --- | --- | --- |
|  | **0-0.2** | **0.2-0.3** | **0.3-0.5** | **0.5-0.8** | **0.8-1.1** | **1.1-1.4** | **1.4-1.9** | **1.9-2.4** | **2.4-3.2** | **3.2-7.7** |
| Fixed Effects | *Output* | *Output* | *Output* | *Output* | *Output* | *Output* | *Output* | *Output* | *Output* | *Output* |
| treatment  VPD  treatment:VPD | F P  0.81 0.54  0.93 0.34  0.76 0.58 | F P  0.33 0.89  2.73 0.10  0.35 0.88 | F P  1.55 0.17  2.55 0.11  0.84 0.52 | F P  0.58 0.71  0.14 0.71  0.29 0.92 | F P  0.49 0.78  0.02 0.89  0.13 0.99 | F P  2.06 0.07  0.02 0.88  1.69 0.14 | F P  2.22 0.05  0.00 0.96  1.45 0.20 | F P  1.10 0.36  2.68 0.10  1.16 0.33 | F P  1.32 0.25  12.77 0.00  1.03 0.40 | F P  4.76 0  12.46 0  4.90 0 |
| Random Effects | *Estimates* | *Estimates* | *Estimates* | *Estimates* | *Estimates* | *Estimates* | *Estimates* | *Estimates* | *Estimates* | *Estimates* |
| σ^2^ | 0.00 | 0.00 | 0.00 | 0.00 | 0.00 | 0.00 | 0.00 | 0.00 | 0.00 | 0.00 |
| τ_00_ | 0.00 _time_ | 0.00 _time_ | 0.00 _time_ | 0.00 _time_ | 0.00 _time_ | 0.00 _time_ | 0.00 _time_ | 0.00 _time_ | 0.00 _time_ | 0.00 _time_ |
| ICC | 0.38 | 0.46 | 0.41 | 0.45 | 0.53 | 0.46 | 0.43 | 0.34 | 0.33 | 0.45 |
| N | 307 _time_ | 342 _time_ | 344 _time_ | 319 _time_ | 343 _time_ | 318 _time_ | 318 _time_ | 279 _time_ | 270 _time_ | 215 _time_ |
| Model | *Values* | *Values* | *Values* | *Values* | *Values* | *Values* | *Values* | *Values* | *Values* | *Values* |
| Observations | 694 | 707 | 707 | 698 | 707 | 705 | 707 | 691 | 705 | 714 |
| *R^2^m* / *R^2^c* | 0.017 / 0.390 | 0.055 / 0.486 | 0.071 / 0.451 | 0.079 / 0.494 | 0.084 / 0.567 | 0.129 / 0.530 | 0.128 / 0.503 | 0.108 / 0.411 | 0.218 / 0.473 | 0.239 / 0.581 |
| Pair differences at 0-0.2 kPa VPD where *p* < 0.05 | | | | | | | | | | |
| - | | | | | | | | | | |
| Pair differences at 0.2-0.3 kPa VPD where *p* < 0.05 | | | | | | | | | | |
| VPD = 0.2700642:  contrast estimate SE df t.ratio p.value  Ambient - eT -0.001905704 0.0006022074 634.77 -3.165 0.0041  Ambient - (eT + eCO2) -0.001163708 0.0004711398 651.84 -2.470 0.0229  Ambient - (Drought + eT + eCO2) -0.002074139 0.0004720036 691.97 -4.394 0.0001  eT - eCO2 0.002167606 0.0006647922 665.20 3.261 0.0035  eT - Drought 0.002107533 0.0006177215 619.83 3.412 0.0026  eCO2 - (eT + eCO2) -0.001425609 0.0005474940 683.66 -2.604 0.0177  eCO2 - (Drought + eT + eCO2) -0.002336040 0.0005442775 695.00 -4.292 0.0001  Drought - (eT + eCO2) -0.001365536 0.0004978627 641.14 -2.743 0.0134  Drought - (Drought + eT + eCO2) -0.002275968 0.0004914713 679.99 -4.631 0.0001  (eT + eCO2) - (Drought + eT + eCO2) -0.000910431 0.0004001346 541.74 -2.275 0.0349 | | | | | | | | | | |
| Pair differences at 0.3-0.5 kPa VPD where *p* < 0.05 | | | | | | | | | | |
| VPD = 0.4347698:  contrast estimate SE df t.ratio p.value  Ambient - eT -0.002540642 0.0006115807 616.18 -4.154 0.0001  Ambient - (eT + eCO2) -0.001076622 0.0005017789 623.44 -2.146 0.0440  Ambient - (Drought + eT + eCO2) -0.002480883 0.0004870667 683.86 -5.094 <.0001  eT - eCO2 0.002799590 0.0006420778 636.24 4.360 <.0001  eT - Drought 0.002923732 0.0006690047 621.91 4.370 <.0001  eT - (eT + eCO2) 0.001464020 0.0005768228 521.00 2.538 0.0191  eCO2 - (eT + eCO2) -0.001335570 0.0005376438 645.81 -2.484 0.0199  eCO2 - (Drought + eT + eCO2) -0.002739831 0.0005244325 690.97 -5.224 <.0001  Drought - (eT + eCO2) -0.001459712 0.0005749678 637.10 -2.539 0.0191  Drought - (Drought + eT + eCO2) -0.002863973 0.0005580610 677.53 -5.132 <.0001  (eT + eCO2) - (Drought + eT + eCO2) -0.001404261 0.0004448709 580.65 -3.157 0.0036 | | | | | | | | | | |
| Pair differences at 0.5-0.8 kPa VPD where *p* < 0.05 | | | | | | | | | | |
| VPD = 0.6455581:  contrast estimate SE df t.ratio p.value  Ambient - eT -0.003476112 0.0006510400 607.24 -5.339 <.0001  Ambient - eCO2 0.001518085 0.0006169031 505.67 2.461 0.0237  Ambient - (eT + eCO2) -0.001434390 0.0006120161 595.76 -2.344 0.0291  Ambient - (Drought + eT + eCO2) -0.002244688 0.0005956916 655.01 -3.768 0.0004  eT - eCO2 0.004994198 0.0006650105 610.37 7.510 <.0001  eT - Drought 0.003690185 0.0007439882 621.32 4.960 <.0001  eT - (eT + eCO2) 0.002041722 0.0005987929 513.33 3.410 0.0013  eT - (Drought + eT + eCO2) 0.001231424 0.0005738712 536.29 2.146 0.0404  eCO2 - (eT + eCO2) -0.002952475 0.0006344794 611.80 -4.653 <.0001  eCO2 - (Drought + eT + eCO2) -0.003762773 0.0006142654 658.64 -6.126 <.0001  Drought - (eT + eCO2) -0.001648463 0.0007171682 622.83 -2.299 0.0298  Drought - (Drought + eT + eCO2) -0.002458761 0.0006954527 655.35 -3.535 0.0009 | | | | | | | | | | |
| Pair differences at 0.8-1.1 kPa VPD where *p* < 0.05 | | | | | | | | | | |
| VPD = 0.9183982:  contrast estimate SE df t.ratio p.value  Ambient - eT -0.003840551 0.0006628829 587.29 -5.794 <.0001  Ambient - (eT + eCO2) -0.001676481 0.0006068097 572.49 -2.763 0.0089  Ambient - (Drought + eT + eCO2) -0.003515131 0.0005805069 638.65 -6.055 <.0001  eT - eCO2 0.004436610 0.0006891684 619.76 6.438 <.0001  eT - Drought 0.003307711 0.0007069194 586.19 4.679 <.0001  eT - (eT + eCO2) 0.002164070 0.0006343268 537.76 3.412 0.0012  eCO2 - (eT + eCO2) -0.002272540 0.0006345174 614.74 -3.582 0.0008  eCO2 - (Drought + eT + eCO2) -0.004111190 0.0006109867 666.72 -6.729 <.0001  Drought - (Drought + eT + eCO2) -0.002982291 0.0006246014 622.78 -4.775 <.0001  (eT + eCO2) - (Drought + eT + eCO2) -0.001838649 0.0005254037 537.00 -3.499 0.0009 | | | | | | | | | | |
| Pair differences at 1.1-1.4 kPa VPD where *p* < 0.05 | | | | | | | | | | |
| VPD = 1.244367:  contrast estimate SE df t.ratio p.value  Ambient - eT -0.005451674 0.0006495139 564.38 -8.393 <.0001  Ambient - (eT + eCO2) -0.001901717 0.0006280821 580.45 -3.028 0.0039  Ambient - (Drought + eT + eCO2) -0.003710874 0.0005715886 653.69 -6.492 <.0001  eT - eCO2 0.006179695 0.0006583011 529.58 9.387 <.0001  eT - Drought 0.004675379 0.0007131485 606.55 6.556 <.0001  eT - (eT + eCO2) 0.003549957 0.0006629111 514.79 5.355 <.0001  eT - (Drought + eT + eCO2) 0.001740800 0.0005940087 522.57 2.931 0.0048  eCO2 - Drought -0.001504316 0.0006644452 576.29 -2.264 0.0299  eCO2 - (eT + eCO2) -0.002629738 0.0006403186 548.83 -4.107 0.0001  eCO2 - (Drought + eT + eCO2) -0.004438895 0.0005848482 618.04 -7.590 <.0001  Drought - (Drought + eT + eCO2) -0.002934579 0.0006378614 669.81 -4.601 <.0001  (eT + eCO2) - (Drought + eT + eCO2) -0.001809157 0.0005790075 571.02 -3.125 0.0031 | | | | | | | | | | |
| Pair differences at 1.4-1.9 kPa VPD where *p* < 0.05 | | | | | | | | | | |
| VPD = 1.6219:  contrast estimate SE df t.ratio p.value  Ambient - eT -0.005831029 0.0006509770 547.81 -8.957 <.0001  Ambient - Drought -0.002082608 0.0006537370 578.45 -3.186 0.0021  Ambient - (eT + eCO2) -0.002687188 0.0006526679 542.38 -4.117 0.0001  Ambient - (Drought + eT + eCO2) -0.003827180 0.0005937441 610.36 -6.446 <.0001  eT - eCO2 0.006828039 0.0007108320 549.30 9.606 <.0001  eT - Drought 0.003748421 0.0006978728 628.16 5.371 <.0001  eT - (eT + eCO2) 0.003143842 0.0006660971 513.22 4.720 <.0001  eT - (Drought + eT + eCO2) 0.002003849 0.0006048666 554.88 3.313 0.0015  eCO2 - Drought -0.003079618 0.0007208763 590.64 -4.272 <.0001  eCO2 - (eT + eCO2) -0.003684198 0.0007055806 534.04 -5.222 <.0001  eCO2 - (Drought + eT + eCO2) -0.004824190 0.0006601547 602.73 -7.308 <.0001  Drought - (Drought + eT + eCO2) -0.001744572 0.0006419350 674.59 -2.718 0.0084 | | | | | | | | | | |
| Pair differences at 1.9-2.4 kPa VPD where *p* < 0.05 | | | | | | | | | | |
| VPD = 2.105878:  contrast estimate SE df t.ratio p.value  Ambient - eT -0.003005990 0.0005607859 554.07 -5.360 <.0001  Ambient - eCO2 0.001669245 0.0005206490 487.45 3.206 0.0027  Ambient - Drought -0.001206996 0.0005603341 589.94 -2.154 0.0475  Ambient - (eT + eCO2) -0.001987101 0.0005474196 552.83 -3.630 0.0007  Ambient - (Drought + eT + eCO2) -0.002002886 0.0005322388 617.28 -3.763 0.0005  eT - eCO2 0.004675235 0.0005643728 554.97 8.284 <.0001  eT - Drought 0.001798994 0.0006004005 635.49 2.996 0.0047  eCO2 - Drought -0.002876241 0.0005657582 595.61 -5.084 <.0001  eCO2 - (eT + eCO2) -0.003656347 0.0005508818 554.61 -6.637 <.0001  eCO2 - (Drought + eT + eCO2) -0.003672131 0.0005365252 616.78 -6.844 <.0001 | | | | | | | | | | |
| Pair differences at 2.4-3.2 kPa VPD where *p* < 0.05 | | | | | | | | | | |
| VPD = 2.754624:  contrast estimate SE df t.ratio p.value  Ambient - eT -0.005729766 0.0005634176 544.04 -10.170 <.0001  Ambient - eCO2 0.002624894 0.0005659051 501.38 4.638 <.0001  Ambient - (eT + eCO2) -0.001984102 0.0005645117 543.10 -3.515 0.0007  Ambient - (Drought + eT + eCO2) -0.002903968 0.0005441763 605.39 -5.336 <.0001  eT - eCO2 0.008354659 0.0005872315 541.94 14.227 <.0001  eT - Drought 0.004502224 0.0006304658 624.39 7.141 <.0001  eT - (eT + eCO2) 0.003745663 0.0005783780 557.19 6.476 <.0001  eT - (Drought + eT + eCO2) 0.002825797 0.0005488475 585.77 5.149 <.0001  eCO2 - Drought -0.003852435 0.0006433449 615.95 -5.988 <.0001  eCO2 - (eT + eCO2) -0.004608996 0.0005880690 540.18 -7.838 <.0001  eCO2 - (Drought + eT + eCO2) -0.005528862 0.0005674826 591.72 -9.743 <.0001  Drought - (Drought + eT + eCO2) -0.001676427 0.0006115575 664.70 -2.741 0.0079 | | | | | | | | | | |
| Pair differences at 3.2-7.7 kPa VPD where *p* < 0.05 | | | | | | | | | | |
| VPD = 4.035747:  contrast estimate SE df t.ratio p.value  Ambient - eT -0.007780428 0.0005903671 527.08 -13.179 <.0001  Ambient - eCO2 0.002149169 0.0005900597 510.79 3.642 0.0006  Ambient - (eT + eCO2) -0.001990300 0.0005998006 537.16 -3.318 0.0015  eT - eCO2 0.009929598 0.0005972443 525.00 16.626 <.0001  eT - Drought 0.008249995 0.0006733572 584.04 12.252 <.0001  eT - (eT + eCO2) 0.005790128 0.0006031681 538.23 9.600 <.0001  eT - (Drought + eT + eCO2) 0.007242103 0.0005911551 593.23 12.251 <.0001  eCO2 - Drought -0.001679602 0.0006745999 568.92 -2.490 0.0178  eCO2 - (eT + eCO2) -0.004139469 0.0006108819 540.05 -6.776 <.0001  eCO2 - (Drought + eT + eCO2) -0.002687495 0.0006034947 606.70 -4.453 <.0001  Drought - (eT + eCO2) -0.002459867 0.0006869935 597.83 -3.581 0.0006  (eT + eCO2) - (Drought + eT + eCO2) 0.001451975 0.0006050061 607.92 2.400 0.0209 | | | | | | | | | | |

**Table S4:** Outputs for the linear mixed-effect model specified as ‘lmer(RWU_VPD_ ~ treatment × SWC + (1|date_and_hour), data = data)’. For details, refer to **S2**. In the ‘fixed effects’ section, ‘output’ shows R outputs generated using the ‘anova’ function. In the ‘random effects’ section, ‘estimates’ refer to variances of residuals and specific random effects, as well as sample numbers. For the model, total observations as well as marginal and conditional *R*-squared values (*R^2^m*, *R^2^c*) are shown. Treatment pair differences at the 5% level of significance are shown. *P*-values were adjusted for multiple testing using false discovery rate correction.

|  | % SWC | | | | | | | | | |
| --- | --- | --- | --- | --- | --- | --- | --- | --- | --- | --- |
|  | **6-11** | **11-14** | **14-18** | **18-20** | **20-22** | **22-24** | **24-26** | **26-29** | **29-32** | **32-39** |
| Fixed Effects | *Output* | *Output* | *Output* | *Output* | *Output* | *Output* | *Output* | *Output* | *Output* | *Output* |
| treatment  SWC  treatment:SWC | F P  2.97 0.03  0.03 0.86  2.65 0.05 | F P  7.78 0  20.65 0  8.20 0 | F P  5.51 0  8.84 0  5.47 0 | F P  0.98 0.43  0.53 0.47  0.92 0.47 | F P  0.90 0.48  1.63 0.20  0.80 0.55 | F P  0.90 0.48  1.63 0.20  0.80 0.55 | F P  4.59 0.00  5.04 0.03  4.52 0.00 | F P  6.33 0.00  3.56 0.06  6.35 0.00 | F P  5.24 0 | F P  0.02 0.90  0.78 0.38  0.01 0.91 |
| Random Effects | *Estimates* | *Estimates* | *Estimates* | *Estimates* | *Estimates* | *Estimates* | *Estimates* | *Estimates* | *Estimates* | *Estimates* |
| σ^2^ | 0.01 | 0.01 | 0.01 | 0.02 | 0.03 | 0.05 | 0.05 | 0.10 |  | 0.12 |
| τ_00_ | 0.07 _time_ | 0.15 _time_ | 0.12 _time_ | 0.08 _time_ | 0.09 _time_ | 0.23 _time_ | 0.28 _time_ | 0.45 _time_ |  | 0.81 _time_ |
| ICC | 0.90 | 0.94 | 0.90 | 0.78 | 0.76 | 0.82 | 0.85 | 0.81 |  | 0.87 |
| N | 569 _time_ | 600 _time_ | 551 _time_ | 609 _time_ | 554 _time_ | 572 _time_ | 507 _time_ | 498 _time_ |  | 438 _time_ |
| Model | *Values* | *Values* | *Values* | *Values* | *Values* | *Values* | *Values* | *Values* | *Values* | *Values* |
| Observations | 729 | 725 | 708 | 724 | 724 | 717 | 701 | 707 | 700 | 612 |
| *R^2^m* / *R^2^c* | 0.027 / 0.900 | 0.044 / 0.947 | 0.031 / 0.902 | 0.028 / 0.785 | 0.059 / 0.775 | 0.045 / 0.832 | 0.037 / 0.855 | 0.037 / 0.822 | 0.036 / 0.029 | 0.001 / 0.874 |
| Pair differences at 6-11 % SWC where *p* < 0.05 | | | | | | | | | | |
| SWC = 9.270391:  contrast estimate SE df t.ratio p.value  (eT + eCO2) - (Drought + eT + eCO2) 0.06423986 0.02406334 230.87 2.670 0.0488 | | | | | | | | | | |
| Pair differences at 11-14 % SWC where *p* < 0.05 | | | | | | | | | | |
| SWC = 12.57181:  contrast estimate SE df t.ratio p.value  eT - Drought -0.1137194 0.04391087 702.05 -2.590 0.0196  Drought - (eT + eCO2) 0.1246251 0.04113775 674.49 3.029 0.0087  Drought - (Drought + eT + eCO2) 0.1196479 0.04002656 624.81 2.989 0.0087 | | | | | | | | | | |
| Pair differences at 14-18 % SWC where *p* < 0.05 | | | | | | | | | | |
| SWC = 15.7947:  contrast estimate SE df t.ratio p.value  Ambient - eT -0.22554165 0.08559213 571.03 -2.635 0.0274  Ambient - Drought -0.21484229 0.08151730 592.38 -2.636 0.0274  Ambient - (eT + eCO2) -0.27547157 0.08344104 602.95 -3.301 0.0153  Ambient - (Drought + eT + eCO2) -0.21582068 0.08251051 606.73 -2.616 0.0274  (eT + eCO2) - (Drought + eT + eCO2) 0.05965089 0.02069964 194.42 2.882 0.0274 | | | | | | | | | | |
| Pair differences at 18-20 % SWC where *p* < 0.05 | | | | | | | | | | |
| SWC = 19.08062:  contrast estimate SE df t.ratio p.value  eT - (eT + eCO2) -0.1175199 0.02878305 370.15 -4.083 0.0004  (eT + eCO2) - (Drought + eT + eCO2) 0.1415303 0.03002400 272.84 4.714 0.0001 | | | | | | | | | | |
| Pair differences at 20-22 % SWC where *p* < 0.05 | | | | | | | | | | |
| SWC = 21.34589:  contrast estimate SE df t.ratio p.value  Ambient - (eT + eCO2) -0.13414902 0.03723413 711.74 -3.603 0.0009  Ambient - (Drought + eT + eCO2) -0.14751749 0.04028987 704.02 -3.661 0.0009  eT - eCO2 0.11648977 0.03880449 711.73 3.002 0.0059  eT - (eT + eCO2) -0.06974814 0.03069831 423.28 -2.272 0.0393  eT - (Drought + eT + eCO2) -0.08311661 0.03416875 423.40 -2.433 0.0289  eCO2 - (eT + eCO2) -0.18623791 0.03509078 690.05 -5.307 <.0001  eCO2 - (Drought + eT + eCO2) -0.19960638 0.03831783 711.86 -5.209 <.0001  Drought - (eT + eCO2) -0.18300120 0.05097974 621.22 -3.590 0.0009  Drought - (Drought + eT + eCO2) -0.19636967 0.05325249 660.13 -3.688 0.0009 | | | | | | | | | | |
| Pair differences at 22-24 % SWC where *p* < 0.05 | | | | | | | | | | |
| SWC = 23.19375:  contrast estimate SE df t.ratio p.value  eT - eCO2 0.2132406 0.07336259 659.06 2.907 0.0189  eCO2 - (Drought + eT + eCO2) -0.2396366 0.06963211 681.06 -3.441 0.0092  (eT + eCO2) - (Drought + eT + eCO2) -0.1197401 0.03924453 314.88 -3.051 0.0186 | | | | | | | | | | |
| Pair differences at 24-26 % SWC where *p* < 0.05 | | | | | | | | | | |
| SWC = 25.13771:  contrast estimate SE df t.ratio p.value  eT - Drought 0.2397802 0.06081904 647.58 3.943 0.0007  Drought - (eT + eCO2) -0.2167792 0.05264684 650.63 -4.118 0.0006  Drought - (Drought + eT + eCO2) -0.1958911 0.05730557 655.86 -3.418 0.0033 | | | | | | | | | | |
| Pair differences at 26-29 % SWC where *p* < 0.05 | | | | | | | | | | |
| SWC = 27.84981:  contrast estimate SE df t.ratio p.value  Ambient - eCO2 -0.1816247 0.06351791 383.25 -2.859 0.0336  eT - Drought 0.2084824 0.06793224 348.38 3.069 0.0336 | | | | | | | | | | |
| Pair differences at 29-32 % SWC where *p* < 0.05 | | | | | | | | | | |
| - | | | | | | | | | | |
| Pair differences at 32-39 % SWC where *p* < 0.05 | | | | | | | | | | |
| - | | | | | | | | | | |

**Table S5**: Outputs for the linear mixed-effect model specified as ‘lmer(fine root property ~ treatment + (1|location/plot) + (1|year/period), data = data)’. For details, refer to **S2**. Response variables include fine root production, traits (SRL = specific root length; SRA = specific root area) and the ratio of fine root- to shoot production (R/S production). In the ‘fixed effects’ section, ‘output’ shows R outputs generated using the ‘anova’ function. In the ‘random effects’ section, ‘estimates’ refer to variances of residuals and specific random effects, as well as sample numbers. For the model, total observations as well as marginal and conditional *R*-squared values (*R^2^m*, *R^2^c*) are shown. Treatment pair differences at the 5% level of significance are shown. *P*-values were adjusted for multiple testing using false discovery rate correction.

|  | Annual production | SRL | SRA | Mean diameter | R/S production |
| --- | --- | --- | --- | --- | --- |
| Fixed Effects | *Output* | *Output* | *Output* | *Output* | *Output* |
| treatment | F P  0.66 0.66 | F P  2.83 0.06 | F P  1.26 0.32 | F P  1.56 0.22 | F P  3.69 0.02 |
| Random Effects | *Estimates* | *Estimates* | *Estimates* | *Estimates* | *Estimates* |
| σ^2^ | 0.01 | 59.82 | 0.01 | 0.00 | 0.06 |
| τ_00_ | 0.00 _plot:location_ | 0.73 _plot:location_ | 0.00 _plot:location_ | 0.00 _plot:location_ | 0.00 _plot:location_ |
|  | 0.00 _location_ | 15.47 _period:year_ | 0.00 _location_ | 0.00 _location_ | 0.01 _period:year_ |
|  | 0.00 _year_ | 2.65 _location_ | 0.01 _period:year_ | 0.00 _period:year_ | 0.00 _location_ |
|  |  | 0.00 _year_ | 0.01 _year_ | 0.01 _year_ | 0.00 _year_ |
| N | 3 _year_ | 3 _period_ | 3 _period_ | 3 _period_ | 3 _period_ |
|  | 26 _plot_ | 3 _year_ | 2 _year_ | 2 _year_ | 3 _year_ |
|  | 7 _location_ | 26 _plot_ | 26 _plot_ | 26 _plot_ | 26 _plot_ |
|  |  | 7 _location_ | 7 _location_ | 7 _location_ | 7 _location_ |
|  |  |  |  |  |  |
| Model | *Values* | *Values* | *Values* | *Values* | *Values* |
| Observations | 67 | 223 | 146 | 146 | 240 |
| *R^2^m* / *R^2^c* | 0.076 / 0.37 | 0.072 / 0.28 | 0.068 / 0.61 | 0.136 / 0.92 | 0.101 / 0.25 |
| Pair differences for annual production where *p* < 0.05 | | | | | |
| - | | | | | |
| Pair differences for SRL where *p* < 0.05 | | | | | |
| contrast estimate SE df t.ratio p.value  Ambient - eT 4.757647 2.149666 18.41 2.213 0.0397  eT - eCO2 -6.563441 2.488361 16.44 -2.638 0.0176  eT - (Drought + eT + eCO2) -5.086973 2.241879 15.80 -2.269 0.0376  eCO2 - Drought 5.962887 2.244362 15.77 2.657 0.0174  Drought - (Drought + eT + eCO2) -4.486419 1.973842 14.42 -2.273 0.0388 | | | | | |
| Pair differences for SRA where *p* < 0.05 | | | | | |
| - | | | | | |
| Pair differences for mean root diameter where *p* < 0.05 | | | | | |
| contrast estimate SE df t.ratio p.value  Ambient - eT -0.02630313 0.01214779 19.92 -2.165 0.0427  eT - eCO2 0.03342382 0.01456380 18.90 2.295 0.0334  eT - (eT + eCO2) 0.02874697 0.01328167 17.85 2.164 0.0442 | | | | | |
| Pair differences for R/S where *p* < 0.05 | | | | | |
| contrast estimate SE df t.ratio p.value  Ambient - eT -0.2309631 0.06675281 17.59 -3.460 0.0231  eT - Drought 0.2419613 0.07434509 17.18 3.255 0.0231  eT - (eT + eCO2) 0.2481931 0.07463204 17.39 3.326 0.0231  eT - (Drought + eT + eCO2) 0.2206940 0.07349573 16.32 3.003 0.0311 | | | | | |

**Table S6**: Summaries for the linear mixed-effect models specified as ‘lmer(*RWU_max_* ~ treatment × production + (1|location/plot) + (1|core_id), data = data)’, and ‘lmer(*RWU_max_* ~ treatment + trait + soil layer + (1|location/plot) + (1|core_id), data = data’. For details, refer to **S2**. Slope effects of predictor variables are indicated by ‘value’. Predictor variables include fine root production and traits (SRL = specific root length; SRA = specific root area). ‘Est’ stands for estimate and ‘SE’ is the standard error. In the ‘fixed effects’ section, ‘output’ shows R outputs generated using the ‘anova’ function. In the ‘random effects’ section, ‘estimates’ refer to variances of residuals and specific random effects, as well as sample numbers. For the model, total observations as well as marginal and conditional *R*-squared values (*R^2^m*, *R^2^c*) are shown.

|  | Root mass density | | | SRL | | | SRA | | | Mean diameter | | |
| --- | --- | --- | --- | --- | --- | --- | --- | --- | --- | --- | --- | --- |
| Predictors | *Est* | *SE* | *p* | *Est* | *SE* | *p* | *Est* | *SE* | *p* | *Est* | *SE* | *p* |
| (Intercept) | 0.45 | 0.12 | **<0.001** | 0.96 | 0.10 | **<0.001** | 1.04 | 0.12 | **<0.001** | 1.31 | 0.13 | **<0.001** |
| value | 9.10 | 2.62 | **0.001** | 0.00 | 0.00 | **0.042** | 0.09 | 0.16 | 0.594 | -1.70 | 0.59 | **0.005** |
| treatment [eT] | 0.11 | 0.19 | 0.564 | 0.04 | 0.10 | 0.667 | 0.06 | 0.14 | 0.667 | 0.09 | 0.13 | 0.476 |
| treatment [eCO2] | -0.04 | 0.19 | 0.853 | -0.06 | 0.11 | 0.584 | 0.00 | 0.14 | 0.989 | -0.02 | 0.13 | 0.864 |
| treatment [Drought] | 0.17 | 0.25 | 0.514 | 0.06 | 0.12 | 0.630 | 0.03 | 0.17 | 0.873 | 0.00 | 0.16 | 0.982 |
| treatment [eT + eCO2] | -0.01 | 0.17 | 0.939 | -0.03 | 0.10 | 0.785 | -0.02 | 0.13 | 0.895 | -0.00 | 0.12 | 0.972 |
| treatment [Drought + eT + eCO2] | 0.08 | 0.18 | 0.636 | -0.07 | 0.10 | 0.442 | 0.02 | 0.13 | 0.888 | -0.00 | 0.12 | 0.972 |
| value × treatment [eT] | -4.43 | 3.61 | 0.221 |  |  |  |  |  |  |  |  |  |
| value × treatment [eCO2] | -3.23 | 4.00 | 0.421 |  |  |  |  |  |  |  |  |  |
| value × treatment [Drought] | -3.11 | 6.06 | 0.608 |  |  |  |  |  |  |  |  |  |
| value × treatment [eT + eCO2] | -1.93 | 3.63 | 0.595 |  |  |  |  |  |  |  |  |  |
| value × treatment [Drought + eT + eCO2] | -5.60 | 3.64 | 0.126 |  |  |  |  |  |  |  |  |  |
| Soil layer [10-20 cm] |  |  |  | -0.43 | 0.05 | **<0.001** | -0.43 | 0.07 | **<0.001** | -0.44 | 0.07 | **<0.001** |
| Soil layer [20-30 cm] |  |  |  | -0.61 | 0.06 | **<0.001** | -0.61 | 0.07 | **<0.001** | -0.62 | 0.07 | **<0.001** |
| Fixed Effects | *Output* | | | *Output* | | | *Output* | | | *Output* | | |
| value  treatment  val:treat  depth | F P  20.32 0.00  0.24 0.93  0.58 0.72 | | | F P  4.17 0.04  0.47 0.80  64.51 0.00 | | | F P  0.29 0.59  0.08 0.99  35.86 0.00 | | | F P  8.19 0.01  0.20 0.95  40.19 0.00 | | |
| Random Effects | *Estimates* | | | *Estimates* | | | *Estimates* | | | *Estimates* | | |
| σ^2^ | 0.15 | | | 0.10 | | | 0.13 | | | 0.12 | | |
| τ_00_ | 0.03 _core_ID_ | | | 0.04 _core_ID_ | | | 0.04 _core_ID_ | | | 0.03 _core_ID_ | | |
|  | 0.00 _plot:location_ | | | 0.00 _plot:location_ | | | 0.00 _plot:location_ | | | 0.00 _plot:location_ | | |
|  | 0.00 _location_ | | | 0.00 _location_ | | | 0.00 _location_ | | | 0.00 _location_ | | |
| N | 11 _plot_ | | | 11 _plot_ | | | 11 _plot_ | | | 11 _plot_ | | |
|  | 4 _location_ | | | 4 _location_ | | | 4 _location_ | | | 4 _location_ | | |
|  | 64 _core_ID_ | | | 64 _core_ID_ | | | 64 _core_ID_ | | | 64 _core_ID_ | | |
| Model | *Values* | | | *Values* | | | *Values* | | | *Values* | | |
| Observations | 228 | | | 229 | | | 158 | | | 161 | | |
| *R^2^m* / *R^2^c* | 0.153 / 0.30 | | | 0.390 / 0.51 | | | 0.332 / 0.45 | | | 0.376 / 0.46 | | |

**REFERENCES**

Bachofen, C., Poyatos, R., Flo, V., Martínez-Vilalta, J., Mencuccini, M., Granda, V., & Grossiord, C. (2023). Stand structure of Central European forests matters more than climate for transpiration sensitivity to VPD. *Journal of Applied Ecology*, *60*(5), 886–897. https://doi.org/10.1111/1365-2664.14383

Bartoń, K. (2024). *MuMIn: Multi-model inference* (1.48.4) [R]. 10.32614/CRAN.package.MuMIn

Bates, D., Mächler, M., Bolker, B., & Walker, S. (2015). Fitting Linear Mixed-Effects Models Using lme4. *Journal of Statistical Software*, *67*(1), 1–48. https://doi.org/10.18637/jss.v067.i01

Bolker, B. (2024). *lme4: Linear mixed-effects models using “Eigen” and S4* (1.1-35.4) [R]. 10.32614/CRAN.package.lme4

Chai, Y., Liu, H., Yu, Y., Yang, Q., Zhang, X., Zhao, W., Guo, L., & Yetemen, O. (2023). Strategies of parameter optimization and soil moisture sensor deployment for accurate estimation of evapotranspiration through a data-driven method. *Agricultural and Forest Meteorology*, *331*(2023), 109354. https://doi.org/10.1016/j.agrformet.2023.109354

Chanzy, A., Gaudu, J.-C., & Marloie, O. (2012). Correcting the temperature influence on soil capacitance sensors using diurnal temperature and water content cycles. *Sensors*, *12*(7), 9773–9790. https://doi.org/10.3390/s120709773

Dubbert, M., Cuntz, M., Piayda, A., Maguás, C., & Werner, C. (2013). Partitioning evapotranspiration – Testing the Craig and Gordon model with field measurements of oxygen isotope ratios of evaporative fluxes. *Journal of Hydrology*, *496*(2013), 142–153. https://doi.org/10.1016/j.jhydrol.2013.05.033

Fox, J. (2023). *car: Companion to applied regression* (3.1-2) [R]. 10.32614/CRAN.package.car

Good, S. P., Soderberg, K., Guan, K., King, E. G., Scanlon, T. M., & Caylor, K. K. (2014). *δ* ^2^ H isotopic flux partitioning of evapotranspiration over a grass field following a water pulse and subsequent dry down. *Water Resources Research*, *50*(2), 1410–1432. https://doi.org/10.1002/2013WR014333

Guderle, M., & Hildebrandt, A. (2015). Using measured soil water contents to estimate evapotranspiration and root water uptake profiles – a comparative study. *Hydrology and Earth System Sciences*, *19*(1), 409–425. https://doi.org/10.5194/hess-19-409-2015

Hothorn, T. (2023). *multcomp: Simultaneous inference in general parametric models* (1.4-25) [R]. 10.32614/CRAN.package.multcomp

Jackisch, C., Knoblauch, S., Blume, T., Zehe, E., & Hassler, S. K. (2020). Estimates of tree root water uptake from soil moisture profile dynamics. *Biogeosciences*, *17*(22), 5787–5808. https://doi.org/10.5194/bg-17-5787-2020

Khalil, M., Sakai, M., Mizoguchi, M., & Miyazaki, T. (2003). Current and Prospective Applications of Zero Flux Plane (ZFP) Method. *Japanese Society of Soil Physics*, *95*(2003), 75–90. https://doi.org/10.34467/jssoilphysics.95.0_75

Lai, Y., Tian, J., Kang, W., Guo, S., Zhou, Y., & He, C. (2023). Estimating evapotranspiration from soil moisture using the improved soil water balance method in cold mountainous areas. *Journal of Hydrology X*, *20*(2023), 100154. https://doi.org/10.1016/j.hydroa.2023.100154

Lenth, R. V. (2024). *emmeans: Estimated marginal means, aka least-squares means* (1.10.2) [R]. 10.32614/CRAN.package.emmeans

Li, Y., Fuchs, M., Cohen, S., Cohen, Y., & Wallach, R. (2002). Water uptake profile response of corn to soil moisture depletion. *Plant, Cell & Environment*, *25*(4), 491–500. https://doi.org/10.1046/j.1365-3040.2002.00825.x

Loheide II, S. P. (2008). A method for estimating subdaily evapotranspiration of shallow groundwater using diurnal water table fluctuations. *Ecohydrology*, *1*(1), 59–66. https://doi.org/10.1002/eco.7

Meeran, K., Ingrisch, J., Reinthaler, D., Canarini, A., Müller, L., Pötsch, E. M., Richter, A., Wanek, W., & Bahn, M. (2021). Warming and elevated CO_2_ intensify drought and recovery responses of grassland carbon allocation to soil respiration. *Global Change Biology*, *27*(14), 3230–3243. https://doi.org/10.1111/gcb.15628

Metze, D., Schnecker, J., Canarini, A., Fuchslueger, L., Koch, B. J., Stone, B. W., Hungate, B. A., Hausmann, B., Schmidt, H., Schaumberger, A., Bahn, M., Kaiser, C., & Richter, A. (2023). Microbial growth under drought is confined to distinct taxa and modified by potential future climate conditions. *Nature Communications*, *14*(1), 5895. https://doi.org/10.1038/s41467-023-41524-y

Nakagawa, S., & Schielzeth, H. (2013). A general and simple method for obtaining *R^2^* from generalized linear mixed-effects models. *Methods in Ecology and Evolution*, *4*(2), 133–142. https://doi.org/10.1111/j.2041-210x.2012.00261.x

Quade, M., Klosterhalfen, A., Graf, A., Brüggemann, N., Hermes, N., Vereecken, H., & Rothfuss, Y. (2019). In-situ monitoring of soil water isotopic composition for partitioning of evapotranspiration during one growing season of sugar beet (Beta vulgaris). *Agricultural and Forest Meteorology*, *266–267*, 53–64. https://doi.org/10.1016/j.agrformet.2018.12.002

R Core Team. (2023). *R: A language and environment for statistical computing.* [Computer software]. R Foundation for Statistical Computing. https://www.R-project.org

Renner, M., Hassler, S. K., Blume, T., Weiler, M., Hildebrandt, A., Guderle, M., Schymanski, S. J., & Kleidon, A. (2016). Dominant controls of transpiration along a hillslope transect inferred from ecohydrological measurements and thermodynamic limits. *Hydrology and Earth System Sciences*, *20*(5), 2063–2083. https://doi.org/10.5194/hess-20-2063-2016

Schelde, K., Ringgaard, R., Herbst, M., Thomsen, A., Friborg, T., & Søgaard, H. (2011). Comparing evapotranspiration rates estimated from atmospheric flux and TDR soil moisture measurements. *Vadose Zone Journal*, *10*(1), 78–83. https://doi.org/10.2136/vzj2010.0060

Verhoef, A., Fernández-Gálvez, J., Diaz-Espejo, A., Main, B. E., & El-Bishti, M. (2006). The diurnal course of soil moisture as measured by various dielectric sensors: Effects of soil temperature and the implications for evaporation estimates. *Journal of Hydrology*, *321*(1–4), 147–162. https://doi.org/10.1016/j.jhydrol.2005.07.039

White, W. N. (1932). *A method of estimating ground-water supplies based on discharge by plants and evaporation from soil: Results of investigations in Escalante Valley, Utah* (USGS Numbered Series 659; Water Supply Paper). U.S. Geological Survey. https://doi.org/10.3133/wsp659A

Yu, C., & Yao, W. (2017). Robust linear regression: A review and comparison. *Communications in Statistics - Simulation and Computation*, *46*(8), 6261–6282. https://doi.org/10.1080/03610918.2016.1202271

Yu, T., Jiapaer, G., Bao, A., Zheng, G., Zhang, J., Li, X., Yuan, Y., Huang, X., & Umuhoza, J. (2022). Disentangling the relative effects of soil moisture and vapor pressure deficit on photosynthesis in dryland Central Asia. *Ecological Indicators*, *137*(2022), 108698. https://doi.org/10.1016/j.ecolind.2022.108698
